# Quantifying Additive Manufacturing Vapor Plumes Using Laser‐Induced Breakdown Spectroscopy, Synchrotron X‐Ray Radiography and Simulations

**DOI:** 10.1002/advs.202513652

**Published:** 2025-12-18

**Authors:** Anna C. M. Getley, Samy Hocine, Junji Shinjo, Chinnapat Panwisawas, Marta Majkut, Alexander Rack, Peter D. Lee, Michael Towrie, Chu Lun Alex Leung

**Affiliations:** ^1^ Department of Mechanical Engineering University College London Torrington Place London WC1E 7JE UK; ^2^ Research Complex at Harwell Rutherford Appleton Laboratory Didcot OX11 0FA UK; ^3^ Next Generation Tatara Co‐Creation Centre (NEXTA) Shimane University Matsue 690‐8504 Japan; ^4^ School of Engineering and Materials Science Queen Mary University of London London E1 4NS UK; ^5^ ESRF – The European Synchrotron Radiation Facility (ESRF) Grenoble 38000 France; ^6^ Central Laser Facility Research Complex at Harwell Rutherford Appleton Laboratory Didcot OX11 0QX UK

**Keywords:** additive manufacturing, alloy composition changes, laser powder bed fusion, laser welding, multi‐physics simulations, preferential vaporization, vapor dynamics, vapor pressures

## Abstract

Understanding vaporization phenomena in laser powder bed fusion (LPBF) additive manufacturing has proven challenging; the links between laser‐induced metal vaporization, rate of elemental loss, and composition irregularities remain unclear. Here, the vapor plume composition and preferential vaporization effect is quantified during LPBF, using in situ 1 kHz laser‐induced breakdown spectroscopy with correlative X‐ray synchrotron radiography, multi‐physics simulations, and energy dispersive X‐ray spectroscopy. It is demonstrated that vaporization increases under keyhole mode, and preferential vaporization causes elemental loss rates of Ni ≈ Fe > Cr > Mo in a Ni‐based superalloy, IN625. It is found that the melt pool temperature (T ≈2300 K) can be approximated by cross‐referencing vapor pressures, and Raoult's law inadequately describes preferential vaporization. Three simulation approaches are compared to show that introducing temperature‐dependent thermophysical properties improves model predictions. The insights into the vapor dynamics of laser‐processed IN625 enhance the understanding of compositional changes and elucidate methods to optimize simulations.

## Graphical abstract

This study presents a novel in situ laser‐induced breakdown spectroscopy system for monitoring metal vapors produced by the intense laser‐metal interactions in laser powder bed fusion additive manufacturing and laser welding. The insights from this new technique reveal changes in alloy compositions and provide the first in situ validation of multi‐physics vaporization simulations, alongside synchrotron X‐ray radiography experiments.

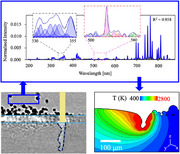

## 1 Introduction

Laser Powder Bed Fusion (LPBF) is a subset of additive manufacturing (AM) that offers unparalleled design freedom and structural complexity for metal manufacturing.^[^ ^1^ ^]^ Components are built by consecutively melting thin metal powder layers to form structures using either, or a combination of, continuous wave or pulse‐width modulated lasers.^[^ ^2^ ^]^ LPBF has a wide range of product applications, including automotive,^[^ ^3^ ^]^ aerospace,^[^ ^4^ ^]^ and biomedical,^[^ ^5^ ^]^ which require repeatable process control and standardized procedures. To implement such protocols, the process and defect formation mechanisms during LPBF must be understood.^[^ ^6^ ^]^ Microstructural features (e.g., pores,^[^ ^7^ ^]^ cracks,^[^ ^8^ ^]^ surface roughness,^[^ ^9^ ^]^ grain structure^[^ ^10^, ^11^ ^]^) and component properties (e.g., fatigue/creep performance^[^ ^12^, ^13^ ^]^) of LPBF components are widely studied in the literature.^[^ ^14^, ^15^ ^]^ However, only a few studies have discussed the effects of the vapor composition and rate of elemental loss on LPBF component quality and alloy composition.^[^ ^16^, ^17^, ^18^ ^]^ Accurate quantitative studies require a high temporal resolution, but are often hindered by significant signal clouding from the chaotic laser‐matter interaction zone.

A vapor plume is emitted during LPBF due to the elevated melt pool temperatures, often approaching or exceeding the material's boiling point. A vaporization‐induced recoil pressure causes a vapor depression – a topological dip in the melt pool surface. At high laser fluence, the recoil pressure creates a long, thin vapor depression which drills down into the melt pool, known as a keyhole. The extent to which species are vaporized is temperature‐dependent; as such, the extent of plume generation in LPBF may vary as a function of melt pool temperature, and may be influenced by the different melting modes, e.g., conduction, transition, and keyhole modes.^[^ ^19^ ^]^ The vapor plume can cause defects, such as track discontinuities, by re‐absorbing some proportion of the laser radiation and reducing the fluence delivered to the melt pool.^[^ ^20^ ^]^ Furthermore, the mixing of the vapor plume with the shielding gas inside the LPBF chamber can create turbulent fluid flows,^[^ ^20^ ^]^ vapor‐induced porosity,^[^ ^17^ ^]^ melt and vapor jets leading to spatter^[^ ^21^ ^]^ and denudation,^[^ ^22^, ^23^ ^]^ leading to cracking or lack‐of‐fusion defects. Preferential vaporization (i.e., the unequal loss of the alloying elements due to differing physical properties)^[^ ^16^, ^24^, ^25^ ^]^ can also occur during LPBF, causing the final part composition to differ from the feedstock material. To minimize such vapor‐induced effects, a better understanding of the volume and composition of vapor under different melting modes is required.

In situ synchrotron X‐ray radiography has been extensively used to observe the printing behavior and melting regimes of LPBF.^[^ ^7^, ^19^, ^26^ ^]^ Whilst synchrotron X‐ray radiography is highly capable of observing solid and liquid behaviors during LPBF with a high spatial (1–5 µm) and temporal resolution (up to 1 µs), it cannot distinguish the much lower‐density vapor plume. The turbulence and characteristics of the plume have been visually analyzed using correlative synchrotron radiography and Schlieren imaging,^[^ ^20^, ^23^, ^27^ ^]^ and similarly, high‐speed optical imaging^[^ ^28^ ^]^ has imaged the plume intensity and trajectory. These studies elucidate the plume flow characteristics but cannot provide compositional analysis of the vapors.

Optical Emission Spectroscopy (OES)^[^ ^29^, ^30^ ^]^ and X‐ray Fluorescence spectroscopy (XRF)^[^ ^31^ ^]^ have been used to examine the chemical composition and improve understanding of the plasma plume above the melt pool in LPBF. The OES apparatus reported to date can qualitatively analyze a limited number of elements (typically 1–2 elements, such as Fe and Cr) at an acquisition speed of up to 100 kHz, whereas the XRF apparatus has a low data acquisition speed (utilizing cumulative measurements over 163 s, i.e., 0.006 Hz) and cannot detect light elements with an atomic mass, Z <11. During LPBF, both techniques may suffer from severe signal clouding due to a large continuum background caused by Bremsstrahlung radiation emitted from the laser‐matter interaction zone, which can limit the spatial resolution for the detection and quantification of individual elements. Most works study the preferential vaporization effect using high‐fidelity multi‐physics simulations, but obtaining quantitative experimental data to verify the model assumptions has proven challenging. These simulation models are often validated using changes in bulk composition rather than vapor compositions.^[^ ^16^, ^17^, ^32^, ^33^ ^]^ The relationships between vapor plume chemistry, preferential vaporization and melting modes in laser welding and AM processes remain unclear.

Laser‐Induced Breakdown Spectroscopy (LIBS) is an alternative chemical analysis method, which examines a plasma ignited by a secondary pulsed light source and utilizes a gated spectrometer to filter out the Bremsstrahlung radiation.^[^ ^34^ ^]^ The spectra only capture the electronic recombination emission signals, substantially improving the signal‐to‐continuum ratio. LIBS can identify all elements, including non‐metals and light metals, in an in situ and online capacity. This provides some advantages compared to directly sampling the melt pool plasma. Here, we develop and apply a novel high‐speed LIBS device for in situ plume composition monitoring during laser welding and LPBF at a rate of 1 kHz. We demonstrate the qualitative and quantitative analytic power of correlative online LIBS combined with in situ high‐speed X‐ray radiography (40 000 frames per second), and its application for understanding the vaporization phenomena during LPBF and laser welding. Our experimental results have been used to verify and validate three different multi‐physics simulation models of vaporization, and to advance our understanding of preferential vaporization and compositional changes in laser material processing.

## 2 Results

### 2.1 In Situ LPBF‐LIBS with Synchrotron X‐Ray Radiography

**Figure 1:**
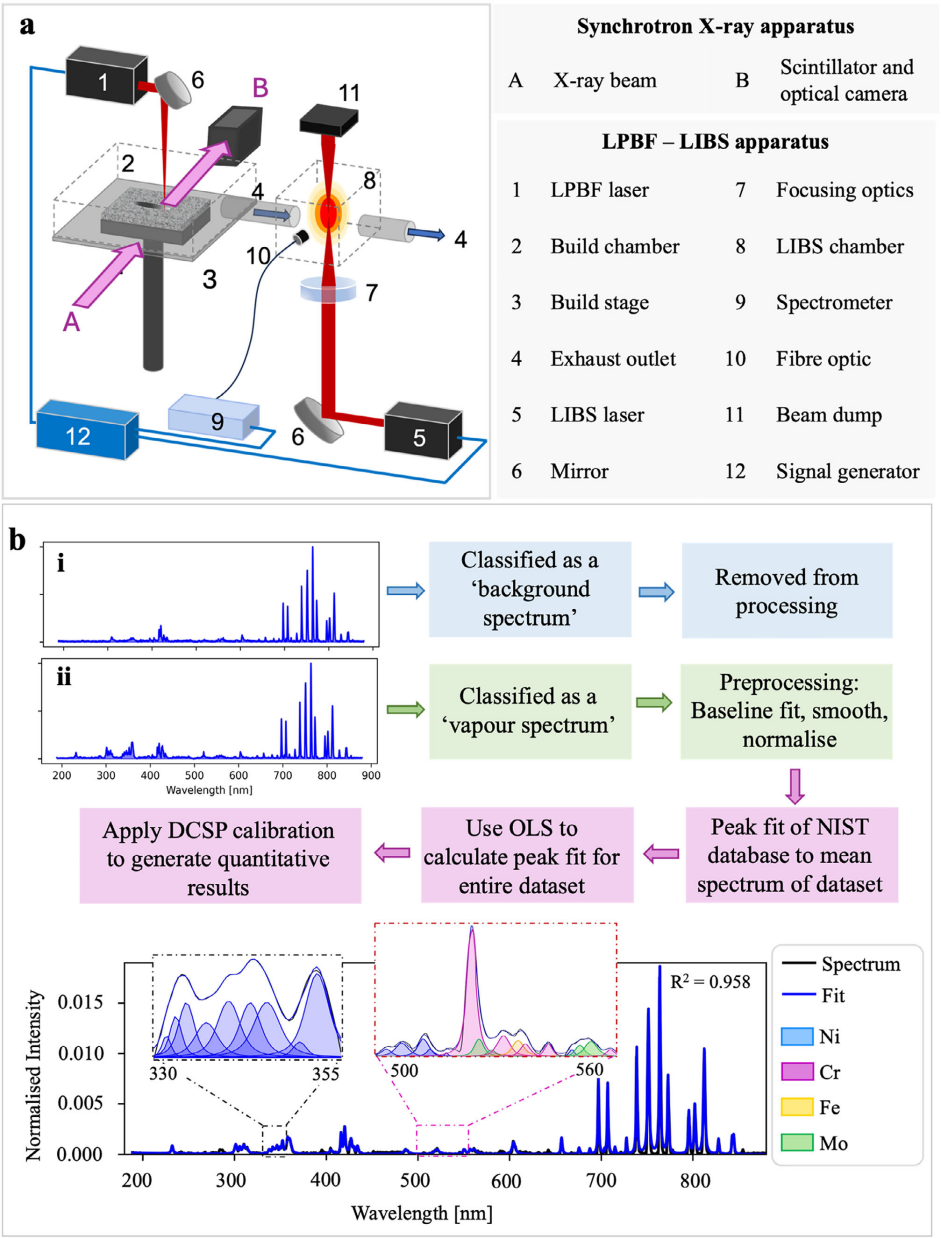
LPBF‐LIBS apparatus, triggering, and quantitative analysis approach. a) A schematic showing the configuration of the LIBS system coupled to the Quad‐ISOPR build chamber and synchrotron X‐ray beam. The characteristics of the LIBS laser pulse emission and plasma lifetime were observed using 1 MHz optical imaging (see Figure S2, Supporting Information). The temporal resolution of the LIBS system (1 kHz) compared to synchrotron X‐ray radiography (40 kHz) allows for total vapor behavior analysis rather than a frame‐by‐frame comparison. b) The LIBS spectra were processed, classified, and deconvoluted using a Python script. Spectra were classified into i) “background” (i.e., argon‐only, no metal vapor present) or ii) “vapor” spectra. Only “vapor” spectra are retained to reduce computation time. The “vapor” spectra were pre‐processed by a baseline fit, smoothing, and normalization before a peak‐finding operation that creates a model of a defined spectral region of interest as a series of overlapping Lorentzian peaks. The model was re‐fitted to each spectrum in the dataset using Ordinary Least Squares (OLS) to obtain peak integrals for every spectrum.

To monitor and quantify the vapor plume composition during LPBF, we designed and built a high‐speed (1 kHz acquisition) LIBS system coupled within a custom Quad‐laser In Situ and *Operando* Process Replicator (Quad‐ISOPR) for LPBF,^[^ ^35^ ^]^ see schematic illustration and data analysis approach in **Figure** **1**, respectively. Additional discussion on the triggering employed to enable high‐speed spectral acquisition without suffering from signal interference is provided in Figure S1 (Supporting Information). The LIBS spectra were pre‐processed, classified, and deconvoluted to obtain the spectral peak integrals (see Figure 1b), before being quantitatively analyzed using a custom Density‐Corrected Single‐Point (DCSP) approach–see details in Methods. To reduce the interference from trace elements, due to the complexity of the multi‐component alloy, our results are normalized to the main constituents of IN625: Ni, Cr, Fe, and Mo.

**Figure 2:**
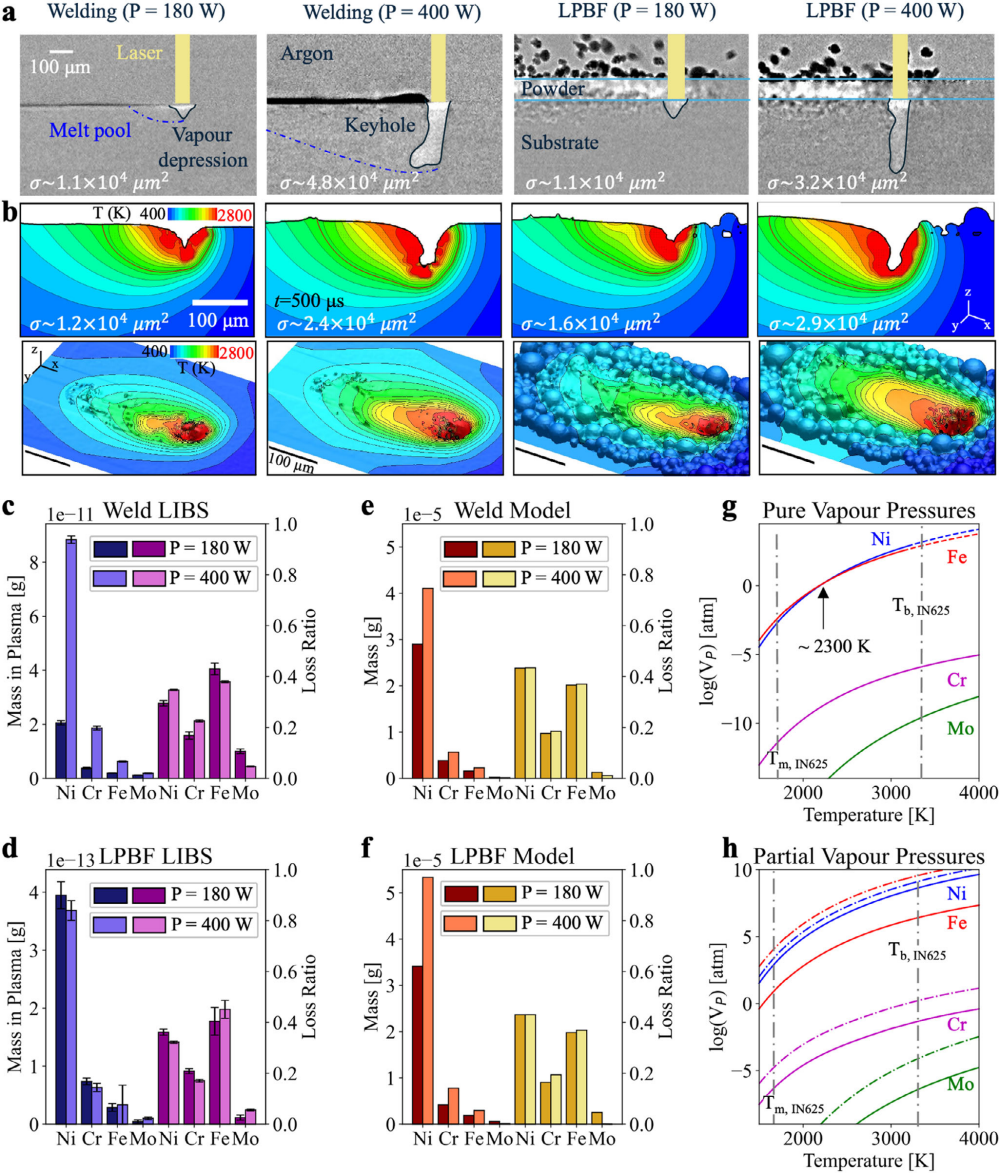
In situ and correlative LPBF‐LIBS with synchrotron X‐ray radiography results. a) Radiographs of the welding and LPBF experiments, demonstrating conduction mode melting at *P *= 180 W, and keyhole mode at *P *= 400 W. The vapor depression surface area (σ) calculated from the experimental data is annotated in each case, estimated as a circular cone surface area (σ = π*AD*/2, for keyhole aperture A and keyhole depth D). b) The 2D (cross‐sectioned in the *y*‐plane) and 3D temperature profiles of the vapor depression zone, estimated by multi‐physics simulation. The simulation vapor depression surface area (σ) is annotated in each case. c,d) LIBS results showing the total vapor composition and loss ratio of (c) the welding, with standard error across all spectra, and (d) the LPBF experiment, with the standard error across three build layers. All quantitative LIBS results were obtained using the DCSP plot approach (see Methods). e,f) Simulation results replicated the total vapor composition and loss ratio of the (e) welding and (f) LPBF prints. g) the vapor pressure of Ni, Cr, Fe, and Mo, plotted using Equation (6), with experimentally determined coefficients for pure liquid metals.^[^ ^41^ ^]^ The melting point (T_m_) and boiling point (T_b_) of IN625 are included for reference. h) The partial vapor pressures of Ni, Cr, Fe, and Mo in an IN625 alloy. The solid lines show the partial pressures as derived using Raoult's law given in Equation (7), whilst the dotted lines give the partial pressures using only the activity coefficient, α_*i*_, and disregarding the mole fraction, χ_*i*_. Numerical results are given in Tables S2–S7 (Supporting Information).

To draw direct parallels between the vapor composition, melting regimes, and vapor depression characteristics, we performed in situ synchrotron X‐ray radiography experiments of IN625 with correlative LIBS composition monitoring. As IN625 behavior has not been previously mapped under in situ radiography conditions, synchrotron X‐ray imaging was employed to ensure that we achieved conduction mode and keyhole mode melting. The radiographs collected during laser welding and LPBF confirm conduction mode melting with a transient but minor vapor depression at a low‐power parameter set, *P* = 180 W and keyhole mode melting at a high‐power parameter set, *P = *400 W, at a constant scan speed, *ν* = 0.5 m s^−1^ (see snapshots in **Figure** **2**a; Videos S1–S4, Supporting Information). Each printing condition was replicated using multi‐physics simulations to identify the melt surface temperatures and evaporative flux – see Figure 2b. We observe a good correlation between the experimental and simulated vapor depression morphology and surface area in each case – indicating a good representation of the vaporization surface. The disparity in keyhole depth in the 400 W case could be owed to the uncertainties in local laser absorptivity and strong temporal fluctuations. An additional discussion on the disparity of simulation vs experimental results, and their associated error, is provided in Discussion S1 (Supporting Information).

To quantify preferential vaporization in IN625, we have defined a loss ratio (see Methods) to measure the rate at which an element is present in the vapor compared to the feedstock substrate and powder compositions. Figure 2c presents the total mass and loss ratio of Ni, Cr, Fe, and Mo in the vapor produced during welding‐LIBS of IN625 (for feedstock compositions see Table S1, Supporting Information). The total mass represents the sum of individual DCSP‐measured masses across the 5 s spectral acquisition time. The total vapor mass increases by 320 ± 7% from 2.75 × 10^−11^ g to 1.15 × 10^−10^ g between conduction to keyhole welding conditions. Prior work shows that keyhole formation at high laser powers^[^ ^36^ ^]^ leads to multiple laser reflections^[^ ^37^ ^]^ inside the keyhole, increasing the total laser absorption. This leads to higher melt pool temperatures and greater metal vaporization. Under both welding conditions, the vapor is mostly composed of Ni, with a small amount of Cr and Fe, followed by a trace level of Mo – similar to the normalized nominal composition of the substrate before welding (≈62% Ni, 23% Cr, 10% Mo, 5% Fe, Goodfellow UK).

The loss ratio for the laser welding experiment suggests a preferential vaporization order of Fe > Ni > Cr > Mo under conduction mode. Under keyhole mode, the loss ratio of Fe and Mo decreases whilst the loss ratio of both Ni and Cr increases, to give a similar preferential vaporization order to the conduction case, but instead with Ni and Fe having a very similar magnitude from a promoted extent of Ni vaporization; hence, given the standard error, the trend becomes Fe ≈Ni > Cr > Mo. Across both welding parameter sets, the loss ratio of Mo is substantially smaller than Ni, Cr, and Fe, indicating a strong preferential vaporization of Ni, Cr, and Fe compared to Mo. Previous work has linked Ni and Cr losses in Ni‐based alloys to surface defects and oxidation.^[^ ^38^ ^]^ As Mo is a solute‐strengthening element in IN625, increased Mo concentration may contribute to greater stiffness.^[^ ^14^ ^]^

Figure 2d presents the total vapor composition masses and loss ratio under LPBF conditions. Similar to the welding cases, the vapors are dominated by Ni in both melting modes, followed by Cr, Fe, and a small amount of Mo. The loss ratio for the LPBF experiment has a preferential vaporization order of Fe ≈Ni > Cr > Mo under conduction mode, and an order of Fe > Ni > Cr > Mo under keyhole mode. The loss ratio magnitudes are similar to the welding experiment, with a slight increase in Fe loss. Under keyhole‐mode LPBF, the loss ratios of Fe and Mo increase, whereas the loss ratios of Ni and Cr decrease.

The total vapor masses are similar between conduction and keyhole mode LPBF. However, the synchrotron X‐ray experiments are susceptible to an error margin arising from small sample volumes. During LPBF, the vapors may condense onto the surrounding cold powder and spatter near the laser‐matter interaction zone, further reducing the sample volume within the LIBS system.^[^ ^26^ ^]^ As such, this effect would be more prominent in LPBF than in welding (see Discussion S2, Supporting Information). To confirm our findings regarding vaporization trends and increased vaporization under keyhole mode melting, the LPBF parameters were repeated over a large printing area (see Validation of LPBF‐LIBS results over large print areas).

### 2.2 Comparison of LPBF‐LIBS with Multi‐Physics Simulation Results

To compare in situ LPBF‐LIBS and synchrotron X‐ray radiography experimental results, we have performed multi‐physics simulations of the processing conditions using the Flint et al.^[^ ^39^ ^]^ model (see Methods). In Figure 2e,f, we have deduced the total vapor masses and loss ratios from the simulation, for the welding and LPBF cases, respectively. The vapor composition in both modeling cases is dominated by Ni and has similar mass fractions to the feedstock material – agreeing with the experimental data (Figure 2c). The vaporized mass is much greater in the model than the experimental data, as we have only sampled the vapor mass contained within the LIBS plasma, i.e., a fraction of the total LIBS chamber volume. The LIBS plasma volume is ≈5 × 10^−5^ cm^3^ (estimated by a cylinder volume π*r* ^2^ *Z_R_*, see Equation (1)), whilst the LIBS chamber volume is 15.1 cm^3^, giving a 10^6^ order of magnitude between the two volumes. The observed mass in the plasma is on the order of 10^−11^ to 10^−13^, as seen in Figure 2c. If we could capture the total vapor volume in the LIBS chamber (i.e., scaling up by 10^6^) the total mass would be on the order of 10^−5^ to 10^−7^ g, which correlates well to the simulation vapor masses.

The order of the loss ratio in both experimental and simulation cases is Ni ≈Fe > Cr > Mo, with similar values for Ni and Fe. In the experimental data, Fe typically dominates slightly over Ni, whilst their order is reversed in the simulation results. This discrepancy is insignificant given the error margin arising from simulation approximations; uncertainties in the temperature‐dependent thermophysical properties (e.g., conductivity, specific heat capacities, density, vapor pressure, surface tension, and viscosity), or physical properties (laser absorptivity, surrounding heat sink properties, alloy mixing, or element size), can cause deviations in the preferential vaporization trends.

### 2.3 Correlation of Preferential Vaporization, Vapor Pressure and Melt Pool Temperature

The vapor pressure – the pressure of a vapor with its condensed phase under thermodynamic equilibrium – is a thermophysical property by which we can infer the likelihood of an element to vaporize from the liquid melt pool at a given temperature. A vapor pressure exists at all temperatures above absolute zero, and it is equal to 1 atm at the elemental boiling point. Figure 2g confirms that Mo has a much lower vapor pressure, i.e., a low vaporization tendency, compared to Ni, Cr, and Fe. The trends of the vapor pressure curve (Ni ≈Fe > Cr > Mo) match well with the loss ratio trends reported in Figure 2c–f. The intersection between Fe and Ni vapor pressure curves at 2300 K indicates that the element with the highest vaporization tendency will shift with temperature.

We hypothesize that cross‐referencing the preferential vaporization tendencies with the vapor pressures of individual alloying elements can be used to approximate the melt pool temperatures. During conduction mode welding, and both LPBF modes, we identify a vaporization rate order of Fe > Ni > Cr, indicating the melt pool temperature is slightly below 2300 K; correlating to the temperatures at which Fe has the highest evaporation flux, whereas Cr has the lowest (except for Mo). Under keyhole mode welding, the loss ratios of Fe and Ni are very similar (Fe ≈Ni > Cr), indicating that the melt pool temperature is approaching the Fe─Ni intersection point at 2300 K.

The LPBF data have a greater difference between Fe and Ni ratios compared to the welding case, indicating that the melt pool temperature was ≈2.4% cooler than the welding pool (see Discussion S3, Supporting Information). This may be attributed to a higher vapor condensation rate compared to welding, due to vapor condensation with the powder layer and spatter, and different laser energy absorption pathways for heating and incorporating the cold powder particles into the melt pool. From the multi‐physics simulations, the average melt pool temperature in the conduction mode welding case is estimated to be 2364 ± 18 K, shifting to 2334 ± 16 K for keyhole mode. In the LPBF simulation, we observe ≈2.2% cooler melt pool temperatures of 2282 ± 20 K and 2312 ± 23 K for conduction and keyhole modes, respectively. In this case, we use the average melt pool temperature to represent the bulk liquid temperature; as the experimental vapor pressures in Figure 2g correlate to temperatures measured from bulk metal liquids, rather than surface temperatures.^[^ ^40^ ^]^ The simulated temperatures are comparable to the LIBS data approximation due to the margin of error in both simulations and experiments.

### 2.4 Comparison of Partial Vapor Pressures to Preferential Vaporization Trends

The vapor pressures of multi‐component alloy elements are often described by Raoult's law; the vapor pressure of an alloy equals the sum of the mole fraction‐ and activity‐weighted individual elemental vapor pressures (see Methods). When the partial vapor pressures are determined in this way (see Figure 2h), there is a wider split between the partial pressures of Ni and Fe – owing to the small mass fraction of Fe in IN625. Raoult's law assumes an ideal mixture, conditional upon the uniformity of adhesive and cohesive forces within the liquid. The ideal gas mixture, however, may not adequately describe the IN625 melt pool, leading to deviations between the expected partial vapor pressures and the experimental observations.

If we negate the mass fraction component of the partial vapor pressure (i.e., only weight the vapor pressure by the elemental activity coefficient), the partial vapor pressures align more closely with the observed preferential vaporization trends, see Figure 2h. Due to the complexity of the inter‐elemental interactions within the molten pool, our results suggest that Raoult's law does not accurately describe the preferential vaporization effect from multi‐component alloy melt pools. Its mole fraction‐weighting assumption is useful for determining the total vapor masses. However, to estimate preferential vaporization, our work suggests that it is more accurate to consider either the pure vapor pressures or the activity coefficient‐weighted vapor pressures, and to disregard the mole fractions of the alloy.

### 2.5 Validation of LPBF‐LIBS Results over Large Print Areas

To confirm our hypothesis on vaporization trends, we have performed LPBF‐LIBS on large (4 × 4 mm^2^) printing areas at a constant scan speed (*v* = 0.5 m s^−1^) and varying laser power (*P* = 180, 300, 400 W) to consolidate the effects of laser power on vaporization rate and preferential vaporization. We validated these results by comparing the chemical composition of the feedstock materials and post‐build tracks using Energy Dispersive X‐ray Spectroscopy (EDS).

**Figure 3:**
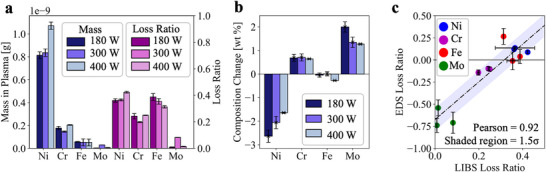
Quantifying the composition of vapors produced during LPBF‐LIBS of large printing areas. a) The LIBS results for large area printing, showing the total mass loss per element and the loss ratio for each species, under 180, 300, and 400 W laser powers. The mass loss of Cr under 300 W processing is further discussed in Discussion S4 (Supporting Information). b) The total change in composition between the EDS of feedstock materials and post‐LPBF print area, calculated using Equation (4), for each laser power. The numerical results are given in Table S8 (Supporting Information). c) A comparison of the LIBS loss ratio to the EDS loss ratio, for all three builds, showing good correlation between the two measures, calculated using Equation (5).

**Figure** **3**a presents the LIBS total vapor mass and loss ratio from the LPBF‐LIBS results for the large 3‐layer builds. Like the synchrotron X‐ray radiography experiments, Ni, Cr, and Fe are readily detected across each print, alongside trace levels of Mo; the vapor composition has similar mass fractions to the feedstock powders and substrates. The total mass of vapor species increases with laser power (see Figure 3a). The total vapor masses (summed over Ni, Cr, Fe, and Mo) are 1.05 ng at 180 W, 1.06 ng at 300 W, and 1.33 ng at 400 W. There is a 27% increase in total vaporized mass between the lowest‐power and highest‐power processing parameters, confirming previous observations of larger vapor plume masses at increased laser powers.^[^ ^20^ ^]^ From 180 to 300 W, we observe the vapor mass to increase by ≈1%; but the vapor mass from 300 to 400 W increases by a much larger extent of 26%. The Flint et al. simulation predicts the 300 W case vapor depression morphology to be in transition mode (see Figure S3, Supporting Information).^[^ ^39^ ^]^ As such, we can conclude that keyhole initiation is a key driver for large increases in vapor masses. The percentage increase in vapor masses between modes remains lower compared to the synchrotron welding experiment, which may be due to the vapor condensation onto cold powders (see Discussion S2, Supporting Information), varying vapor trajectories as indicated by Bitharas et al.,^[^ ^20^ ^]^ and larger vaporization at low laser powers compared to the weld – due to increased laser absorptivity from the powder layer in LPBF.

To clarify the preferential vaporization effect described in Figure 2, we also report the loss ratio over large LPBF print areas in Figure 3a. We observe preferential vaporization of Fe, Ni, and Cr relative to Mo, under all processing conditions. The large print area confirms that Mo exhibits the smallest vaporization effect in IN625. This is further confirmed by EDS results in Figure 3b, which demonstrated consistent increases in the mass fraction of Mo in the post‐build track compared to the feedstock material across all three builds. Our work also agrees with a previous study showing compositional gains in Mo in LPBF‐produced SS316L.^[^ ^42^ ^]^

Compared to the synchrotron experiment, the large prints experience hotter melt pools due to a greater residual heating effect from the overlap of bi‐directional scan paths. The preferential vaporization order is Fe > Ni > Cr > Mo for the 180 W build, indicating a sub‐2300 K melt temperature; but as the laser power increases, the loss ratio of Fe decreases whilst Ni and Cr loss ratios increase. This replicates the trends observed during the synchrotron X‐ray experiment and further confirms the validity of the Flint et al.^[^ ^39^ ^]^ model for simulating vaporization losses. Under higher laser powers of 300 and 400 W, we observe a vaporization tendency shift to Ni > Fe > Cr > Mo. This corresponds to a temperature exceeding 2300 K from the elemental vapor pressures, i.e., the region of the vapor pressure plots where Ni and Fe deviate more strongly as temperature increases (see Figure 2g). This effect extends further as the laser power increases from 300 to 400 W. These estimates align with prior work^[^ ^36^ ^]^ that predicted a melt pool temperature range of 3000–3500 K during the LPBF of IN625 at an input Linear Energy Density (LED) of 750 J m^−1^ (where LED = laser power/laser scan speed). By comparison, the LED in this work is 600 J m^−1^ at 300 W and 800 J m^−1^ at 400 W. We report a larger composition change for Ni and Mo at lower laser power of 180 W compared to that of 400 W results, see Figure 3b. This is due to the higher LED reducing the preferential vaporization effect by inducing a stronger vaporization response across all alloying species, i.e., as the temperature moves toward the boiling point of IN625, the vapor pressures of Ni, Fe, Cr, and Mo become much closer in magnitude (see Figure 2g).

To compare the measurements of preferential vaporization by in situ LIBS and ex situ EDS, we have devised a loss ratio equation for EDS bulk measurements, as given in Equation (5). For the EDS loss ratio, a negative result indicates a relative gain in an elemental weight percent compared to the feedstock compositions (averaged over the feedstock powders and substrate), whilst a positive ratio – similar to the LIBS loss ratio – indicates a relative loss in weight percent of that element. Figure 3c shows a strong positive correlation between the LIBS and EDS loss ratios with a Pearson coefficient of 0.919. We can confirm that a small loss ratio in the LIBS data was correlated to a gain in bulk composition, and vice versa. The preferential vaporization trends seen in the vapor spectral data are mirrored by the post‐print changes in bulk composition measured by EDS – further validating the vapor composition monitoring and quantification results powered by LIBS.

### 2.6 Comparison of Different Multi‐Physics Simulation Approaches

**Figure 4:**
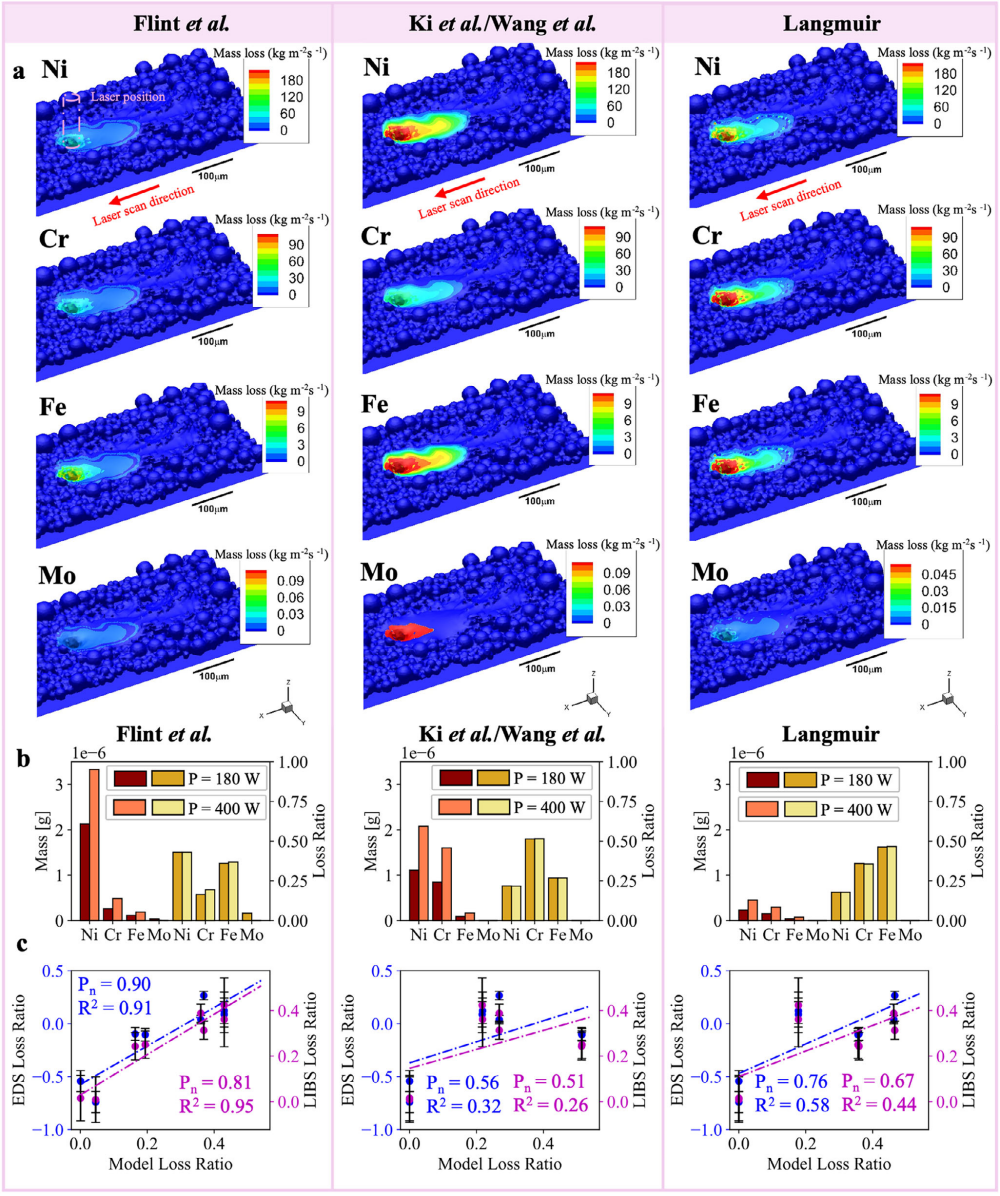
Simulation results and comparison of different approaches. a) The simulation results for evaporative flux of Ni, Cr, Fe, and Mo during LPBF printing using three different simulation approaches (Flint et al., Ki et al./Wang et al., and Langmuir). b) The total vaporized masses and loss ratios calculated from each of the models. All numerical data is given in Tables S6 and S7 (Supporting Information). c) Comparison of the experimental EDS and LIBS loss ratios to the simulated loss ratios.

To fully investigate the correlation of simulation, LPBF‐LIBS, and ex situ characterization, we performed multi‐physics simulations using two other methodologies; the Ki et al./Wang et al. method and the Langmuir method (see Methods).^[^ ^43^, ^44^ ^]^ We have compared all three simulations to the EDS and LIBS loss ratios after printing from the large area LPBF experiments, to discuss the validities and merits of these simulation approaches. **Figure** **4**a shows the results for the evaporation flux of Ni, Cr, Fe, and Mo for all three modeling approaches.

Figure 4b gives the total vapor mass and loss ratios from each simulation using Equation (3). The Flint et al. model predicts the largest vaporization losses, with a total vaporized mass of 2.55 to 4.01 µg for 180 and 400 W, respectively. The Ki et al./Wang et al. model predicts a slightly smaller mass but of similar magnitude: 2.05 to 3.84 µg dependent on laser power. Conversely, the Langmuir model predicts much smaller vaporization losses of 0.42 to 0.82 µg. The results from the Flint et al. and Ki et al./Wang et al. models corroborate with the total vaporized mass from the synchrotron X‐ray experimental data, which is expected to be on the order of 0.1 to 10 µg. The Langmuir model seems to result in lower estimates of the vapor masses comparatively.

By comparing the LIBS results to the Flint et al. model in Figure 2c,d, it is apparent that the elemental loss ratios are of similar trend and magnitude, with preferential vaporization orders of roughly Ni ≈Fe > Cr > Mo. In contrast to the Flint et al. model, the Ki et al./Wang et al. model indicates Cr > Fe > Ni > Mo, whilst the Langmuir simulation predicts Fe > Cr > Ni > Mo. To rationalize the correct preferential vaporization order, we plot each of the simulated loss ratios against the EDS and LIBS loss ratios in Figure 4c. The comparison shows the Flint et al. model results have the strongest correlation to both vapor loss ratios and post‐build measurements, as per the highest Pearson coefficient (*P_n_*) of 0.81 and R^2^ value of 0.95 (see Methods).

The Langmuir model calculates the evaporative flux by solving the Maxwell–Boltzmann velocity distribution for free atoms over the melt pool surface. Both the Flint et al. model and the Ki et al./Wang et al. model are extensions of the Langmuir model; the former incorporates the latent heat of vaporization, and ratios of the densities and temperatures between the gas and liquid state. By comparison, the latter defines jump conditions for atoms between the substrate layer, Knudsen layer, and outer air layer to determine the populations in each layer. All three modeling approaches correctly identify the low Mo vaporization tendency, however the trends between Ni, Cr, and Fe evaporative losses vary between the simulations. This confirms our hypothesis that the partial pressures described by Raoult's law inadequately describe the preferential vaporization tendencies for IN625. Both Langmuir and Ki et al./Wang et al. models rely extensively on the partial vapor pressure to determine evaporative flux. By comparison, the Flint et al. model incorporates further temperature‐dependent properties, e.g., latent heat of vaporization, density of states, and liquid‐gas temperature ratios, resulting in a more robust model that does not suffer from the effects of non‐ideal mixing upon partial vapor pressures in the liquid state of a multi‐component alloy.

## 3 Discussion

Our synchrotron X‐ray imaging, in situ LIBS, ex situ EDS, and multi‐physics modeling results have quantified and elucidated vaporization losses and preferential vaporization tendencies during the laser welding and LPBF of IN625. We report the first high‐speed composition monitoring and quantification of vapor plumes during laser welding‐LIBS and LPBF‐LIBS at 1 kHz, under conduction and keyhole melting modes. The vaporization rates typically increase under keyhole mode compared to conduction mode. This is due to the higher laser fluence and multiple laser reflections off the undulating keyhole wall, greatly increasing the depression surface area and the temperature of the liquid‐vapor interface, driving a stronger vaporization effect.

We have introduced the loss ratio to explain the preferential vaporization effect during laser processing of IN625, demonstrating that Fe and Ni are lost at a greater rate than Cr and Mo due to differing physical properties. By studying the preferential vaporization effect, we can also approximate the melt pool temperatures in IN625 using pure vapor pressure plots. The mole‐weighted component of Raoult's law for partial pressures is found to be inadequate for describing preferential vaporization trends in laser welding or LPBF; instead, the relative loss of individual elements is more accurately described by either the pure vapor pressures or the elemental activity coefficient.

Out of the three modeling approaches (Flint et al., Ki et al./Wang et al., and Langmuir), the Flint et al. modeling results prove to be the closest match to the experimental data for the total vapor mass (accounting for sample volume), the preferential vaporization trends, and the melt pool temperature. The robustness of the Flint et al. model can be attributed to the inclusion of temperature‐dependent thermophysical properties (e.g., latent heat of vaporization, density of states, and liquid‐gas temperature ratios), which reduced the reliance of the model on the Raoult's law assumption of an ideal gas mixture.

Finally, our work demonstrates that vapor composition monitoring and quantification using LIBS is instrumental to understanding both vapor characteristics during laser metal processing and advancing our understanding in determining the best approaches to simulating laser‐induced metal vaporization. Ultimately, we can use this powerful tool to fine‐tune laser processing parameters to achieve optimal post‐build chemistry, microstructural properties, and part performance.

## 4 Experimental Section

### Quad‐ISOPR LPBF Process Replicator with LIBS System

The Quad‐ISOPR consisted of a 1070 nm fiber laser (RenAM 500Q scan head), a build chamber, a high‐speed coaxial optical imaging camera (Photron Nova S12), and the LIBS system, embedded within a certified Class I laser enclosure (EN 60825:1). The laser beam was focused onto the LPBF‐printing plane with a 4σ spot diameter of 80 µm. The LPBF environmental build chamber included a pressure sensor, an automated powder hopper, and a high‐resolution *z*‐stage actuator. The Quad‐ISOPR was custom‐built to accommodate synchrotron radiography experiments; as such, the build chamber contained two glassy carbon windows to allow X‐ray transmission. The operational principles of the Quad‐ISOPR were detailed by Hocine et al.^[^ ^35^ ^]^ The build chamber was purged continuously with a 3 L min^−1^ argon shielding gas flow for 2 min prior to printing to avoid oxidation during the build. The chamber pressure was maintained at + 10 kPa prior to printing.

Vapors generated during laser welding or LPBF were carried by the shielding gas, and exited the build chamber via the exhaust, before being transported toward the in situ LIBS system; analysis of the error introduced by condensation at the exhaust is included in Discussion S7 (Supporting Information). The LIBS system consisted of an air‐tight secondary chamber directly attached to the exhaust manifold of the LPBF build chamber, which was equipped with custom CaF_2_ optical windows. The LIBS plasma was generated by a Nd: YAG pulsed laser (Bright Solutions Inc., Italy: 1064 nm, 1 ns pulse length, 3.12 mJ pulse energy, 1000 Hz frequency, linear 100:1 polarization, *M* ^2^  ≥  3), which was collimated, then focused, through two plano‐convex lenses to the center of the secondary chamber. Two lenses were installed to correct for the beam quality. LIBS sampling was acquired continuously for 5 s after printing commenced, at a rate of 1,000 Hz.

The plasma light was collected through a collimating lens (*f* = 25 mm) and a 400 µm core fiber optic directly attached to the secondary chamber, normal to the laser propagation direction. The other end of the fiber optic cable was fed into a broadband spectrometer (Ocean FX UV–vis ES, Ocean Insight, UK: spectral resolution Δλ = 1.69 nm FWHM, CMOS detector). The spectrometer was calibrated over a spectral range of 187–872 nm using an Ar─Hg lamp (Ocean Insight HG‐2, UK) – see Figure S4 (Supporting Information). The LIBS spectra were recorded over a 10 µs integration time with a 1.5 µs gate delay after the LIBS laser pulse. The LIBS system was triggered by the Quad‐ISOPR via a fully integrated control system (PandABox, Quantum detector, UK), ensuring synchronization with laser melting, see the corresponding triggering diagram in Figure S1 (Supporting Information).

### Welding‐LIBS and LPBF‐LIBS Experiments

A commercial nickel‐based alloy (Inconel 625 or IN625) is selected as the material for this study. The main alloying constituents of IN625 were all high‐Z transition elements (Ni, Cr, Fe, and Mo), allowing good visibility under EDS. The IN625 feedstock materials also contain a significant weight percentage of Nb (3.46 wt.% in the substrate, and 3.54 wt.% in the powder) – however, the NIST LIBS database does not contain Nb data, therefore Nb cannot be reliably resolved in this study. A comment was provided on the likely behavior of Nb‐based on the SEM‐EDS and simulation results – in Discussion S5 (Supporting Information).

Two geometries were printed: i) 0.18 × 4 mm^2^ two‐line bi‐directional tracks under synchrotron radiography conditions, and ii) 4 × 4 mm^2^ bi‐directional hatched tracks in laboratory conditions for the large print experiments. Both geometries employed an 80 µm hatch spacing. For the synchrotron radiography experiments, bi‐hatch welding was conducted on an IN625 (Goodfellow, UK) substrate for a low‐power (*P* = 180 W and *ν* = 0.5 m s^−1^) and high‐power parameter set (*P* = 400 W and *ν* = 0.5 m s^−1^). In the LPBF synchrotron experiments, three layers of IN625 powder (Carpenter Additive, UK, particle size distribution of 15–45 µm) were printed atop an IN625 substrate using the same low‐ and high‐power parameter sets. The selected layer thickness was 60 µm. LPBF‐LIBS was performed during the printing of each layer, and used a fixed scan speed to reduce the number of variables that could affect the vaporization behavior.

For the 4 × 4 mm^2^ bi‐directional hatches, three layers of IN625 powder were printed atop an IN625 substrate, performing LPBF‐LIBS for each layer. The selected layer thickness was 60 µm. The low‐power (*P* = 180 W, *ν* = 0.5 m s^−1^) and high‐power (*P* = 400 W, *ν* = 0.5 m s^−1^) parameter sets were repeated, and performed an additional mid‐power parameter set (*P = *300 W, *ν* = 0.5 m s^−1^).

### In Situ Synchrotron X‐Ray Radiography

To capture the melting and keyhole dynamics, the Quad‐ISOPR rig at the ID19 beamline at ESRF – the European Synchrotron Radiation Facility, Grenoble, France (proposal number: ME1573) was operated – see the beamline settings detailed by Hocine et al.^[^ ^35^ ^]^ The LPBF process was illuminated by a polychromatic hard X‐ray beam produced by a U32 undulator, with a photon energy range of 20–50 keV, and a mean energy of ≈30 keV. A series of propagation‐based X‐ray phase contrast radiographs were converted to visible light via a 250 µm LuAg:Ce scintillator, and recorded using a high‐speed Photron SA‐Z camera (40 000 fps with a 18.8 µs exposure time) with a 4.315 µm pixel resolution over a field of view (FOV) of 4.5 × 2.2 mm^2^. The triggering of the Quad‐ISOPR, LIBS system, and X‐ray shutter were synchronized through the Quad‐ISOPR control panel. The LIBS system was housed on the right‐hand side of the Quad‐ISOPR build chamber, to prevent any interference with the synchrotron X‐ray beam path. The LIBS spectra were collected in a time‐resolved manner – for brevity, the compositional findings were presented. To resolve the keyhole morphology, the images were flat‐field corrected and background‐subtracted as detailed by Hocine et al.^[^ ^35^ ^]^

### Spectral Deconvolution and Analysis

Each LIBS spectrum was pre‐processed by applying a baseline removal using a local‐minima linear approximation for every 70 points. The filtered spectrum was smoothed by a Savitsky‐Golav filter, before being normalized by dividing the spectrum by its sum integral, a.k.a. total light normalization.^[^ ^45^ ^]^ The pre‐processing pipeline classified each spectrum as a “background” (argon only, i.e., no metal vapor was contained within the sample volume, see Figure 1b i) or a “vapor” (e.g., metal peaks, Figure 1b ii) spectrum, based on the peak locations. To improve computational efficiency, only “vapor” spectra were retained for peak deconvolution. Deconvolution^[^ ^46^, ^47^, ^48^, ^49^ ^]^ was first performed on the mean of the spectral dataset. For each region of interest in the spectrum, a Lorentzian curve was assigned to each peak, defining peak locations by cross‐referencing with the NIST LIBS database,^[^ ^50^ ^]^ in the region using the *lmfit* library^[^ ^51^ ^]^ (see Figure 1b), creating a model of the spectrum as a matrix of overlapping Lorentzian peaks. The Lorentzian peaks model was refitted to each spectrum in the dataset using an Ordinary Least Squares calculation, to obtain the individual peak integrals. Using this method, the spectral dataset was analyzed at a rate of ≈100 spectra s^−1^. For qualitative analysis, the deconvoluted data was matched against the NIST LIBS database^[^ ^50^ ^]^ to identify the elements present within the plasma volume.

### LIBS Calibration and Quantitative Analysis

For quantitative analysis, a hybrid approach combining the One‐Point Calibration (OPC) method^[^ ^52^ ^]^ and the calibration curve method was used;^[^ ^53^, ^54^ ^]^ due to the complexity of calibrating for an alloy sample in the vapor state (see Discussion S6 Supporting Information for further information).^[^ ^55^, ^56^ ^]^ Solid‐state LIBS was first performed on the feedstock IN625 substrate placed inside the LIBS chamber (pulse energy 3.12 mJ, repetition rate 1000 Hz) – see Figure S5 (Supporting Information). The OPC coefficients were calculated using the solid‐state LIBS spectra to obtain the solid‐state plasma density.^[^ ^52^, ^57^ ^]^ From the LIBS spectra, a list of suitable calibration peaks was identified for each element (non‐overlapping, minimal self‐absorption and < 400 nm to avoid interference from argon emissions); these peaks are {Ni: 229.0, 231.9, 234.7 and 352.9 nm}, {Cr: 267.0 and 288.0 nm}, {Fe: 252.6 nm}, {Mo: 390.7 nm}.

A single‐point calibration curve of density‐corrected sampled mass was plotted against the summed intensity (*I_peaks_*) for each element, referred to as the Density‐Corrected Single‐Point (DCSP) plot. The sampled mass was calculated from the plasma density as:

(1) $$m_{i}=\pi r^{2}Z_{R}\left(N_{s,i}^{I}+N_{s,i}^{II}\right)\frac{M_{R,i}}{N_{A}}$$

where *m_i_* is the mass of element *i* in grams, $N_{s,i}^{I}$ and $N_{s,i}^{II}$ are the number of species of the I and II state of element *i* derived from the Saha–Boltzmann *y*‐intercepts (in mm^−3^) equal to the plasma density, *M*_*R*, *i*_ is the molar mass of element *i* in g mol^−1^, *N_A_* is Avogadro's number, *r* is the LIBS laser spot radius and *Z_R_* is the Rayleigh length of a laser. The sum of the observed peak intensities per element was plotted against the known masses of the SRM elements with a linear fit (*I_peaks_* =  *n*(*m_i_*) + *c*). The DCSP plots are given in Figure S6 (Supporting Information), and coefficients (*n*,  *c*) are given in Table S9 (Supporting Information). To extract the semi‐quantitative results from the deconvoluted data, the integrals of all peaks were identified that match the *I_peaks_* list for each element (Ni, Cr, Fe, Mo). The sum of the integrals per element was extracted and calculated the masses using the DCSP plot coefficients as:

(2) $$m_{i}^{u}=\frac{\left(I_{peaks}^{u}-c\right)}{n}\;$$

for each spectrum *u* with unknown masses $m_{i}^{u}$, and spectral peak integrals $I_{peaks}^{u}$.

After quantitative analysis to determine the elemental masses in the vapor, the preferential vaporization effect was compared using a normalized Loss Ratio (LR), calculated as:

(3) $$LR_{LIBS,i}=\frac{\left(m_{i}^{sum}/\chi_{i}.M_{r}\right)}{\sum_{i}\left(m_{i}^{sum}/\chi_{i}.M_{r}\right)}\;$$

where *LR*_*LIBS*, *i*_ is the LIBS loss ratio of element *i*, $m_{i}^{sum}$ is the mass of the element *i* in the vapor – in this work, the sum over the dataset is used; χ_*i*_ is the mass fraction of element *i* in the feedstock alloy (dimensionless), and *M_r_* is the molecular mass of the feedstock material (in g mol^−1^). Therefore, χ_*i*_.*M_r_* is equal to the molar mass of each element *i* in the alloy. The term $(m_{i}^{avg}/\chi_{i}.M_{r})\;$ represents the elemental loss per mole of feedstock material (in mol), and the result was normalized by the sum of all $\left(m_{i}^{avg}/\chi_{i}.M_{r}\right)$ over all elements in the feedstock. Therefore, the normalized loss is presented as a dimensionless ratio, summing to 1 over all elements. It should be noted that the vapor masses obtained using this methodology are best regarded as relative measurements between samples as opposed to absolute mass values.

### Ex Situ Bulk Composition Analysis

The chemical composition of IN625 powder, substrates, and printed parts was measured by Scanning Electron Microscope Energy Dispersive X‐ray Spectroscopy (SEM‐EDS). The EDS maps were constructed with a secondary electron imaging (SEI) mode at an accelerated voltage of 20 keV and a 54–60 µm spot size over a 1‐hour live acquisition time. The compositions were normalized to include only the main alloying constituents (Ni, Cr, Fe and Mo) to avoid the results being skewed by changes in trace elements, see Table S1 (Supporting Information). All EDS measurements were re‐scaled using calibration curves constructed using EDS measurements of four metal alloy Standard Reference Samples (SRM) from the National Institute of Standards and Technology (NIST) (see Methods S1, Supporting Information).

After measurement by EDS, the relative composition change (Δ_*i*_) for element *i* is reported as:

(4) $$\Delta_{i}=C_{T,i}-\;\frac{1}{2}\left(C_{P,i}+C_{S,i}\right)\;$$

where *C*_*T*, *i*_ is the weight percentage of element *i* in the LPBF build [wt.%], *C*_*P*,*i*_ is the weight percentage of element *i* in the feedstock powder [wt.%] and *C_S_*,*_i_* is the weight percentage of element *i* in the feedstock substrate [wt.%]. As only three layers are processed, an average of the powder and substrate composition was assumed as the feedstock composition to account for the dilution effect. To compare the preferential vaporization effect in the bulk measurement, a loss ratio was calculated for the bulk in a similarly to Equation (3), as:

(5) $$LR_{\mathrm{EDS},i}=\frac{-\Delta_{i}\;/0.5\left(C_{P,i}+C_{S,i}\right)}{\sum_{i}\left|\left(-\Delta_{i}\;/0.5\left(C_{P,i}+C_{S,i}\right)\right)\right|}\;$$

Compared to the LIBS loss ratio, the EDS loss ratio has a sign change; as strong vaporization of an element leads to a negative Δ_*i*_ as the element is lost from the bulk – therefore the negative sign ensured a loss in bulk composition relates to a positive loss ratio, i.e., high vaporization rate. This allowed the LIBS and EDS loss ratios to be comparable. Similar to the LIBS loss ratio, the term (− Δ_*i*_ /0.5(*C*_*P*,*i*_ +  *C_S_*,*_i_*)) represented the change in weight percentage compared to the feedstock weight percentage. Finally, the result was normalized by the magnitude of (− Δ_*i*_ /0.5(*C*_*P*,*i*_ +  *C_S_*,*_i_*)) over all elements to get the dimensionless ratio. The EDS loss ratio can produce both negative and positive loss ratios, relating to a net gain and a net loss of an element, respectively.

### Vapor Pressure Equations

The vapor pressures of the pure metal elements were plotted using the following equation:

(6) $$log\;\left(P\right)=-\frac{A}{T}+B+Clog\left(T\right)+10^{-3}DT$$

where P is the vapor pressure (mm Hg), T is the temperature (K) and A, B, C and D are experimentally determined coefficients given by Brandes et al.,^[^ ^41^ ^]^ listed in Table 1, except for those denoted by ^†^ – which were obtained from Mondal et al.^[^ ^58^ ^]^

**Table 1:** The coefficients A, B, C, and D for Equation (6). The coefficients are representative of liquid metals at elevated temperatures and are valid for the given temperature ranges only.

Element	State	A	B	C	D	Temperature range [K]
**Ni**	Solid	22500	13.6	−0.96	0	298 – 1726
**Ni**	Liquid	22400	16.95	−2.01	0	1726 – 3183
**Cr**	Solid	20680	14.56	−1.31	0	298 – 2130
**Cr ^†^**	Liquid	21790	15.86	−2.42	−0.024	1400 – 3525
**Fe**	Solid	21080	16.89	−2.14	0	298 – 1809
**Fe**	Liquid	19710	13.27	−1.27	0	1809 – 3133
**Mo**	Solid	34700	11.66	−0.236	−0.145	298 – 2893
**Mo ^†^**	Liquid	40260	43.96	−10.43	0.565	2200 – 5760

The vapor pressures of the elements contained in a multi‐component metallic alloy can be approximated by Raoult's law; the vapor pressure of an alloy equals the sum of the mole fraction‐weighted individual elemental vapor pressures:

(7) $$V_{p}=\sum_{i}\chi_{i}\alpha_{i}V_{i}$$

for mole fraction χ_*i*_, activity coefficient α_*i*_ and pure vapor pressure *V_i_* of element *i* in the alloy. The total vapor pressure is the sum of (χ_*i*_α_*i*_ *V_i_*) over all elements in the alloy composition.

### Multi‐Physics Simulation Methods

Multi‐physics modeling based on computational fluid dynamics (CFD) was used to track the temperature field under LPBF and welding conditions. The model was detailed extensively by Shinjo et al.^[^ ^59^ ^]^ and Zhang et al.,^[^ ^60^ ^]^ see Methods S2 (Supporting Information) for further detail. The method included fluid flow dynamics of molten metal, melting, solidification, liquid/solid/gas interface tracking, heat transfer, surface tension with the Marangoni effect and laser ray tracing. The numerical scheme was based on the Constrained Interpolation Profile (CIP) method, in which a third‐order polynomial fitting was used for flow variables and their derivatives to assure the numerical accuracy. The physical properties such as viscosity and thermal conductivity were retrieved from Shinjo et al.^[^ ^61^ ^]^ The grid resolution was set to 4 µm, by which the temperature and velocity fields can be well reproduced under the present settings.^[^ ^59^, ^60^ ^]^ The numerical method was detailed in previous work and not repeated here.^[^ ^59^, ^60^ ^]^

The vapor mass loss was re‐calculated using the simulation temperature data with the vaporization models described below. Each model determined the evaporative flux losses in kg s^−1^, which were then multiplied by the total printing time for a single track (8 ms at ν = 0.5 m s^−1^) to obtain the total vaporised mass given in Table S6 (Supporting Information). In the following section, the nuances of the three vaporization models used to determine the evaporative flux in this study are detailed.^[^ ^25^, ^39^, ^43^, ^44^, ^62^, ^63^, ^64^, ^65^, ^66^ ^]^

### Multi‐Physics Simulation Methods—Langmuir Model

The Langmuir model has been used widely in welding and LPBF vaporization calculations.^[^ ^25^, ^62^, ^63^, ^64^ ^]^ The original Langmuir model assumes that the atoms move freely under equilibrium. The Maxwell–Boltzmann probability distribution of the velocity for the free motion of an atom was used and the mass flux (kg m^−2^ s^−1^) was obtained as:

(8) $$j_{i}=p_{i}\;\sqrt{\frac{m_{i}}{2\pi k_{B}T}}=p_{i}\;\sqrt{\frac{m_{i}N_{A}}{2\pi \tilde{R}T}}=p_{i}\;\sqrt{\frac{M_{i}}{2\pi \tilde{R}T}}$$

where *p_i_* is partial vapor pressure (Pa) of chemical species *i*, *m_i_* (kg) is the mass of the atom, $k_{B}=\tilde{R}/N_{A}\;$ is the Boltzmann constant, $\tilde{R}$ is the universal gas constant (8.314 J mol^−1^ K^−1^), *T* is top surface temperature of the melt pool (K), *N_A_* is the Avogadro number (6.02×10^23^ mol^−1^) and *M_i_* is atomic mass (kg mol^−1^). If *j_i_* is converted into (mol m^−2^ s^−1^), and with the assumption of equilibrium where the evaporation and condensation coefficients are identical and the accommodation factor is introduced to include the ambient pressure effect, then:

(9) $$J_{i}=\frac{j_{i}}{M_{i}}=\frac{\lambda p_{i}a_{i}}{\sqrt{2\pi M_{i}\tilde{R}T}}$$

where *λ* is the accommodation factor and set as 0.8 and *a* is the activity.^[^ ^64^ ^]^ The activity *a* was calculated by Thermo‐Calc, which is provided in Figure S7 (Supporting Information).^[^ ^67^ ^]^ The mass loss rate, ${\dot{m}}_{i}$, of species *i* (kg m^−2^ s^−1^) is:

(10) $${\dot{m}}_{i}=J_{i}M_{i}$$

### Multi‐Physics Simulation Methods—Flint et al. Model

This was an extended version of the Langmuir model.^[^ ^39^ ^]^ The evaporative flux for chemical species *i* (kg m^−2^ s^−1^) was given as:

(11) $${\dot{m}}_{i}=\frac{6\alpha_{l,i}\sqrt{\frac{M_{i}}{2\pi \tilde{R}T_{v,i}}\rho_{l}\rho_{g}L_{v,i}}}{b_{d}\left(\rho_{l}-\rho_{g}\right)}\frac{T-T_{v,i}}{T_{v,i}}$$

where α_*l*,*i*_ is the liquid fraction of chemical species *i*. The subscripts *l* and *g* denote liquid and gas, respectively, and *v* denotes vaporization. *L*_*v*,*i*_ is the latent heat (J kg^−1^), and ρ denotes the density of each state. A thermal fluid dynamics framework was applied to multi‐component substrates experiencing fusion and vaporization state transitions. It is clear that $\sqrt{\frac{M_{i}}{2\pi \tilde{R}T_{v,i}}}\rho_{g}L_{v,i}$ is equivalent to $j_{i}=p_{i}\;\sqrt{\frac{M_{i}}{2\pi \tilde{R}T}}$ in the Langmuir model (Equation (8)). *b_d_* is a scale factor to represent the ratio of surface area and volume above the vaporising surface.^[^ ^65^ ^]^ It can be given by *b_d_* = (2 − σ)/2σ  where σ is the evaporation coefficient, which is identical to the condensation coefficient, and σ ≈ 0.01 for 1 atm.^[^ ^65^ ^]^

### Multi‐Physics Simulation Methods—Ki et al. and Wang et al. Model

The original modeling idea dates back to Knight et al.,^[^ ^43^, ^44^, ^66^ ^]^ by which the outer layers of the Knudsen layer were considered, and the connection with the ambient air was formulated.^[^ ^43^, ^44^ ^]^ A schematic of the model, and full derivation, are given in Methods S2 (Supporting Information). The basis of the model involved simulating the different vapor layers above the substrate, and the jump conditions required for an atom to be promoted from the substrate to the outer air vapor layer (which has a height sufficient for that atom to be described as vapor). Following Ki et al.,^[^ ^43^ ^]^ the formulations are shown below. The subscript for chemical species *i* is omitted here for clarity.

Over the bottom Knudsen layer, a vapor layer exists between the Knudsen layer and the outer air layer. For the ambient air pressure conditions of 1 atm, the flow was assumed to be subsonic in the outer edge of the Knudsen layer. Since the Knudsen layer is very thin (and not in the continuum regime), it can be viewed as a discontinuity and the jump conditions between layers are:

(12) $$\frac{p_{s}}{p_{a}}=\frac{p_{v}}{p_{a}}/\frac{p_{v}}{p_{s}}\;$$

(13) $$\frac{p_{s}}{p_{v}}=\frac{\rho_{s}T_{s}}{\rho_{v}T_{v}}\;$$

(14) $$\frac{a_{v}}{a_{a}}=\frac{\sqrt{\gamma_{v}R_{v}T_{v}}}{\sqrt{\gamma_{a}R_{a}T_{a}}}\;$$

for ratios of pressure, *p*, and temperature *T*, specific heats γ, and assuming Mach number <1 for subsonic interactions. The subscript *a* refers to the outer air layer, *s* refers to the substrate interface with the Knudsen layer, *v* is the interface between the Knudsen layer and vapor layer. *R_v_* is the gas constant (in J kg^−1^ s^−1^) and *a* is the speed of sound. The net mass loss rate (evaporation–condensation) is therefore:

(15) $$\dot{m}=\rho_{S}\sqrt{\frac{RT_{S}}{2\pi }-}\rho_{v}\sqrt{\frac{RT_{v}}{2\pi }}\beta F^{-}\left(\tilde{m}\right)$$

where:

(16) $$\beta =\frac{2\left(2{\tilde{m}}^{2}+1\right)\sqrt{T_{v}/T_{s}}-2\sqrt{\pi }\tilde{m}}{F^{-}+\sqrt{T_{v}/T_{s}}G^{-}}$$

(17) $$F^{-}=\sqrt{\pi }\tilde{m}\left(-1+\mathrm{erf}\left(\tilde{m}\right)\right)+\exp\left(-{\tilde{m}}^{2}\right)$$

(18) $$G^{-}=\left(2{\tilde{m}}^{2}+1\right)\left(1-\mathrm{erf}\left(\tilde{m}\right)\right)-\frac{2}{\sqrt{\pi }}\tilde{m}\exp\left(-{\tilde{m}}^{2}\right)$$

(19) $$\tilde{m}=\frac{u_{v}}{\sqrt{2R_{v}T_{v}}}=\sqrt{\frac{\gamma_{v}}{2}}M_{v}$$

where *u_v_* is the velocity, γ_*v*_ is the ratio of specific heats, and *M_v_* is the Mach number.

To solve the jump condition, typically the Clapeyron‐Clausius relation is used. This formulation is consistent with the mass flux (kg m^−2^ s^−1^) of the Langmuir model shown in Equation (8), because:

(20) $$\dot{m}=\rho_{S}\sqrt{\frac{RT_{S}}{2\pi }}=\frac{p_{S}}{RT_{S}}\sqrt{\frac{RT_{S}}{2\pi }}=p_{S}\frac{1}{\sqrt{2\pi RT_{S}}}=p_{S}\sqrt{\frac{M}{2\pi \tilde{R}T_{S}}}$$

using *p*  =  ρ*RT*, and molar mass *M*. The Ki et al./Wang et al. model was based on this idea, see derivations in the original references.^[^ ^43^, ^44^ ^]^

### Model Comparison Methods

To evaluate the different model performances, the correlation was determined between the loss ratio predicted by the model (using Equation (3)) and the EDS loss ratio measured from the large print areas (calculated using Equation (5)), using the Pearson correlation coefficient, *P_n_*, calculated with the *SciPy* library:^[^ ^68^ ^]^

(21) $$P_{n}=\frac{\sum \left(x-m_{x}\right)\left(y-m_{y}\right)}{\sqrt{\sum {\left(x-m_{x}\right)}^{2}\sum {\left(y-m_{y}\right)}^{2}}}$$

where *x* is the set of model loss ratios with a mean value of *m_x_*, and *y* is the set of EDS loss ratios with a mean value of *m_y_*. The coefficient can take a value of − 1  ≤ *P_n_*  ≤ 1, with −1 indicating a strong negative correlation, 0 being no correlation, and +1 being a strong positive correlation.

### Melt Pool Temperature Calculations and Comparison Methods

The average melt pool temperature is given by:

(22) $$T_{pool,avg}=\frac{1}{V_{pool}}\int_{pool}T\;dV_{pool}$$

where the temperature *T* is integrated over the pool volume, *V_pool_*.

## Conflict of Interest

Chu Lun Alex Leung, Mike Towrie, and Anna Getley are co‐inventors on UK patent 2500841.8 detailing the LIBS system, filed January 2025 by University College London Business Ltd. The remaining authors declare no competing interests.

## Author Contributions

C.L.A.L., M.T., and P.D.L. conceptualized the project and acquired funding. A.C.M.G. and C.L.A.L. designed the experiments, with contributions from M.T. and S.H. A.C.M.G. performed the experiments, with support from S.H. and C.L.A.L. S.H. designed and built the Quad‐ISOPR rig. M.M. and A.R. supported beamtime experiments at ESRF. C.P. and J.S. designed and executed the modeling. A.C.M.G. completed the data analysis, results interpretation, and data visualization with contributions from C.L.A.L., M.T., P.D.L., S.H., C.P., and J.S. A.C.M.G., and C.L.A.L. drafted the manuscript, with contributions and reviewing from P.D.L., M.T., J.S., C.P., S.H., and A.R.

## Supporting information



Supporting Information

Supporting Information

Supporting Information

Supporting Information

Supporting Information

## Data Availability

The data that support the findings of this study are available in the supplementary material of this article.
