# Supplementary material for: Quantifying Additive Manufacturing Vapor Plumes Using Laser‐Induced Breakdown Spectroscopy, Synchrotron X‐Ray Radiography and Simulations
Source: Adv Sci (Weinh). 2025 Dec 18;13(12):e13652. doi: 10.1002/advs.202513652 (PMC12948290; doi:10.1002/advs.202513652)
Supplement: Supplementary file 1 — Supporting Information [file ADVS-13-e13652-s003.docx]

# Quantifying additive manufacturing vapour plumes using laser-induced breakdown spectroscopy, synchrotron X-ray radiography and simulations

Anna C.M. Getley ^a, b^ *, Samy Hocine ^a, b^, Junji Shinjo ^c^, Chinnapat Panwisawas ^d^, Marta Majkut ^e^, Alexander Rack ^e^, Peter D. Lee ^a, b^, Michael Towrie ^f^, Chu Lun Alex Leung ^a, b^ *

* Corresponding authors: [alex.leung@ucl.ac.uk](mailto:alex.leung@ucl.ac.uk) and [anna.getley.16@ucl.ac.uk](mailto:anna.getley.16@ucl.ac.uk)

^a^ Department of Mechanical Engineering, University College London, Torrington Place, London, UK

^b^ Research Complex at Harwell, Rutherford Appleton Laboratory, Didcot, UK

^c^ Next Generation Tatara Co-Creation Centre (NEXTA), Shimane University, Matsue, Japan

^d^ School of Engineering and Materials Science, Queen Mary University of London, London, UK

^e^ ESRF – The European Synchrotron Radiation Facility (ESRF), Grenoble, France

^f^ Central Laser Facility, Research Complex at Harwell, Rutherford Appleton Laboratory, Didcot, UK

# Supplementary Information

## Supplementary Figures


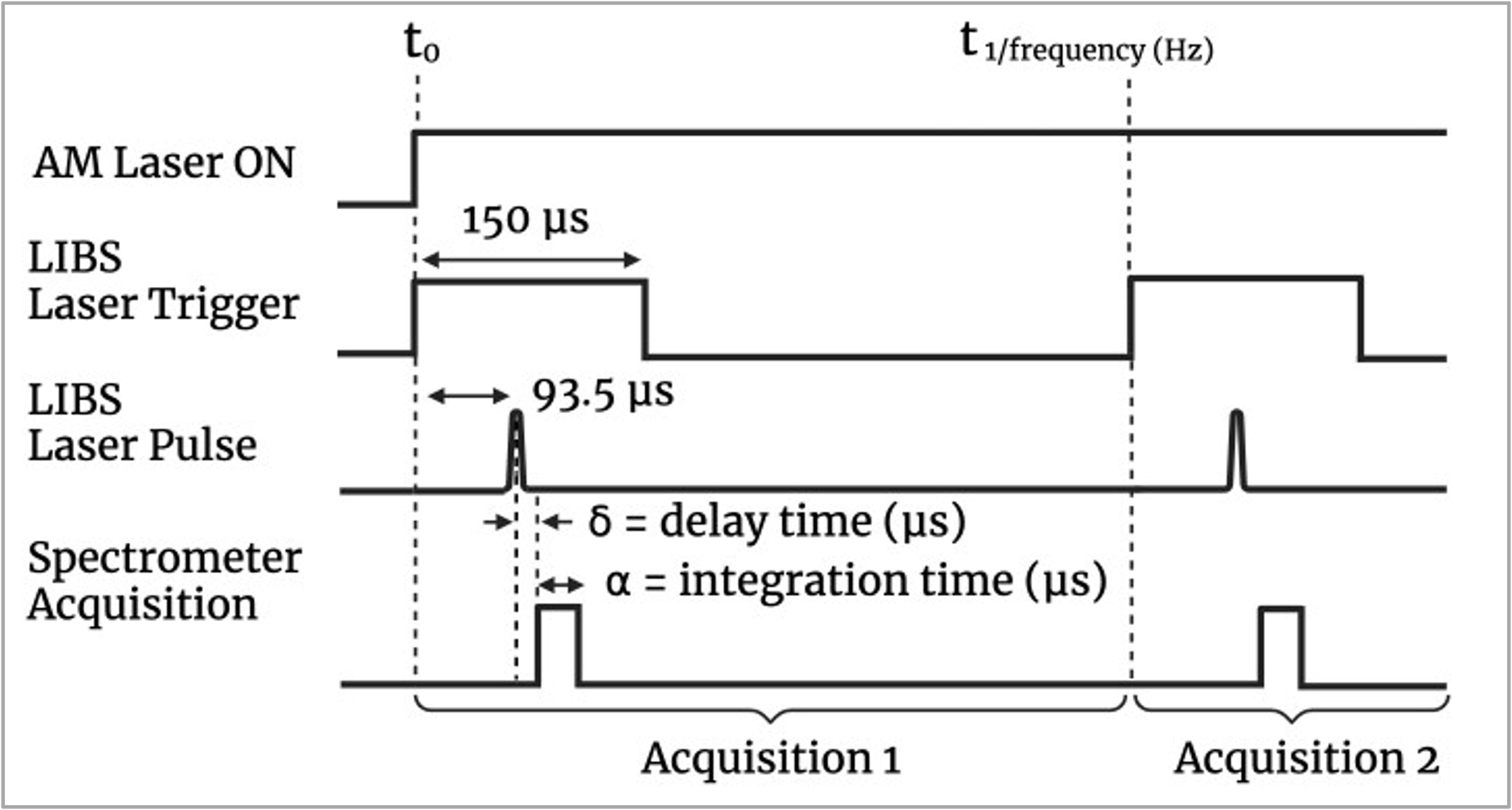


### Supplementary Figure 1: The LPBF-LIBS triggering diagram

The triggering system is designed to enable high-speed LIBS acquisition without suffering from spectral interference caused by the graybody continuum or overlapping plasma ignition. The delay time, δ = 1.5 μs, is applied to gate the continuum light from detection by the spectrometer. The spectrometer integration time, α = 10 μs. After LIBS sampling, the vapour and argon gas flows exit through the LPBF exhaust system to the side of the build chamber.


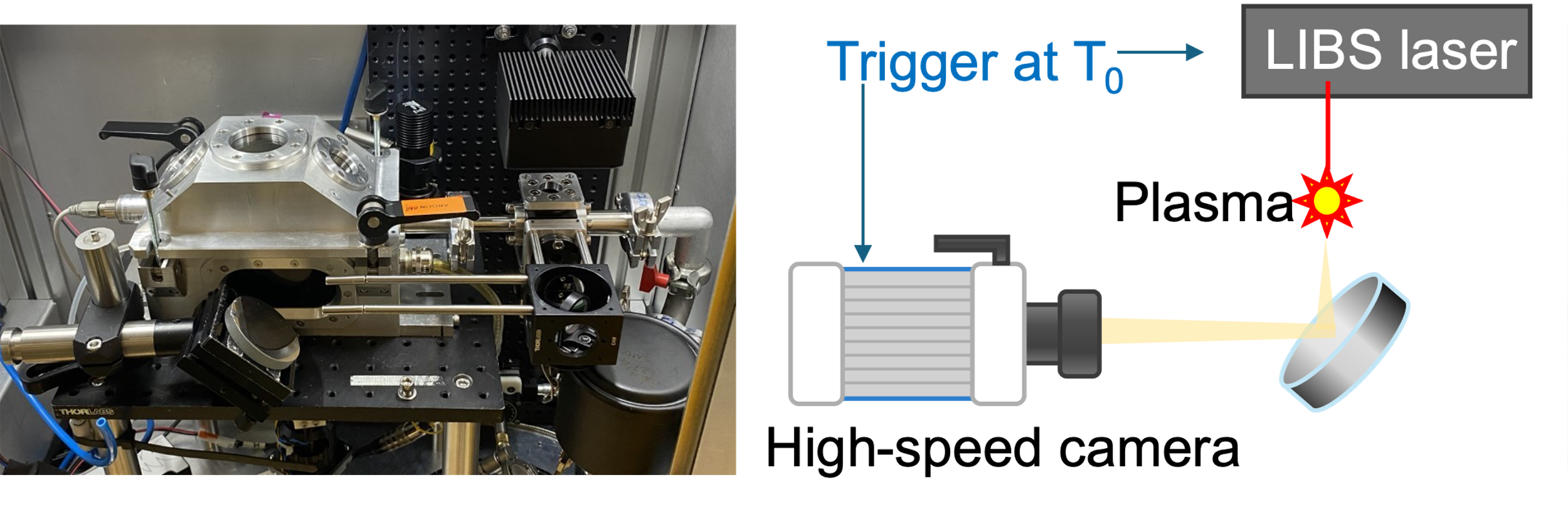


### Supplementary Figure 2: High-speed optical imaging apparatus used to observe the laser-induced plasma behaviour

The LIBS plasma was projected onto an aluminium mirror and reflected towards a 1 MHz optical camera (Photron FASTCAM NOVA S12, Photron, Japan) coupled to the scan head of the Quad-ISOPR. The LIBS laser and optical camera were triggered simultaneously via two identical TTL outputs. Using this apparatus, the plasma lifetime was observed to be 10 µs.


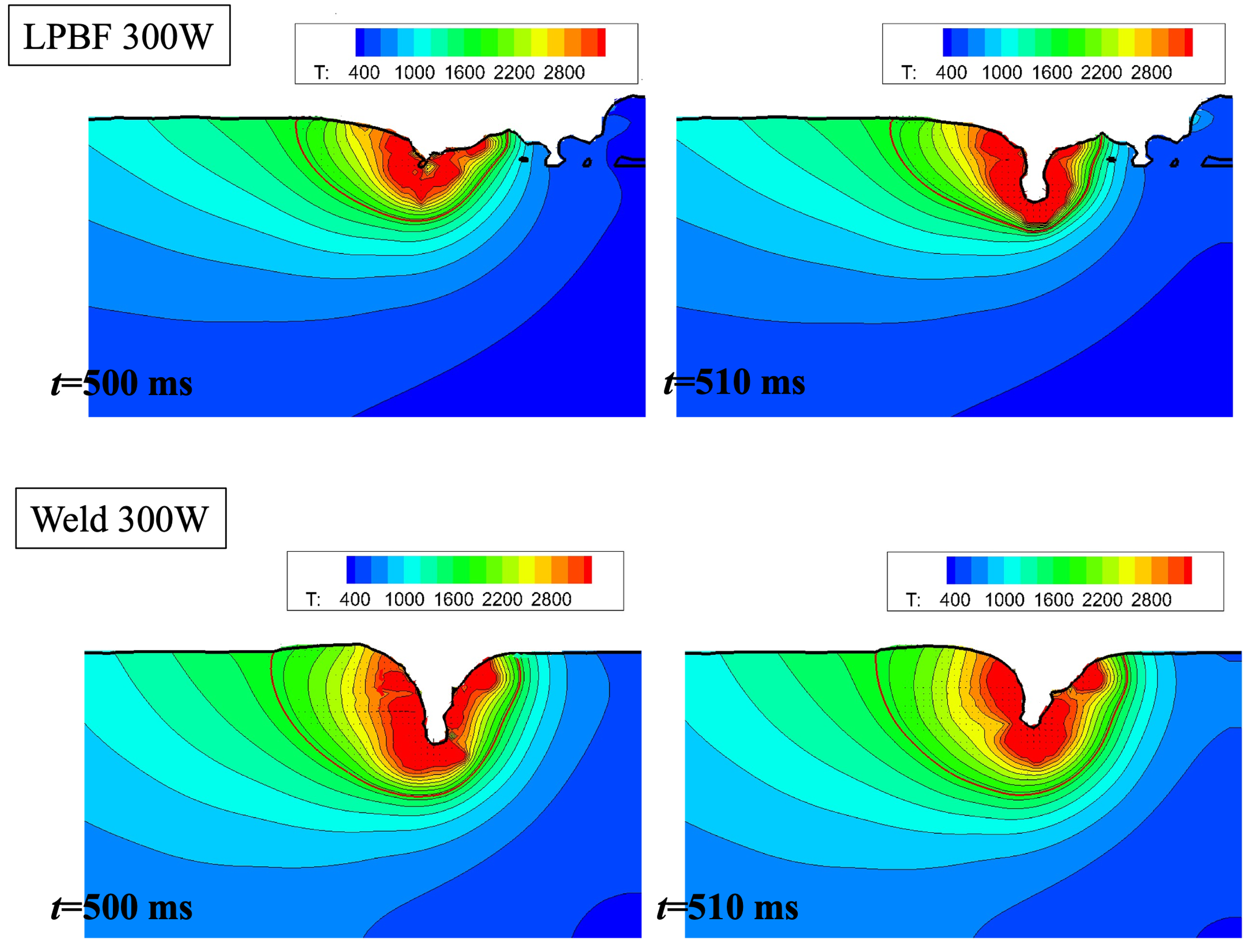


### Supplementary Figure 3: The vapour depression predicted by the Flint *et al.* simulation for LPBF performed at *P =* 300 W and *v =* 0.5 m s^-1^. ^1^

We observe that the geometry of the vapour depression fluctuates within the transition melting regime under the simulation condition of *P =* 300 W and *v* *=* 0.5 m s^-1^. We define this condition as transition mode, especially as the true vapour depression is likely to be slightly deeper than the predicted one (see **Supplementary Discussion 1**), though both keyholes exhibit a similar surface area.


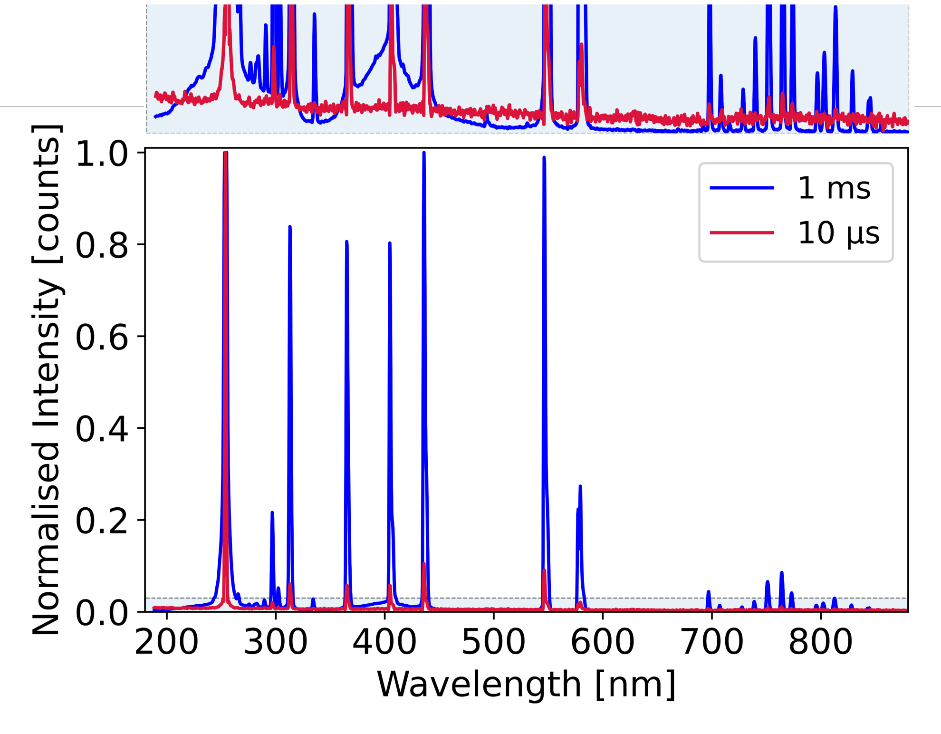


### Supplementary Figure 4: The response of the spectrometer to a calibration lamp (Ocean Insight, Ar-Hg HG-2) over different integration times

The short integration time used in the LIBS experiments (10 µs) shows a strong preference for some peaks and increased signal noise. The relative intensity of peaks can fluctuate with different integration times; further supporting the use of multiple peaks for calibration curves.


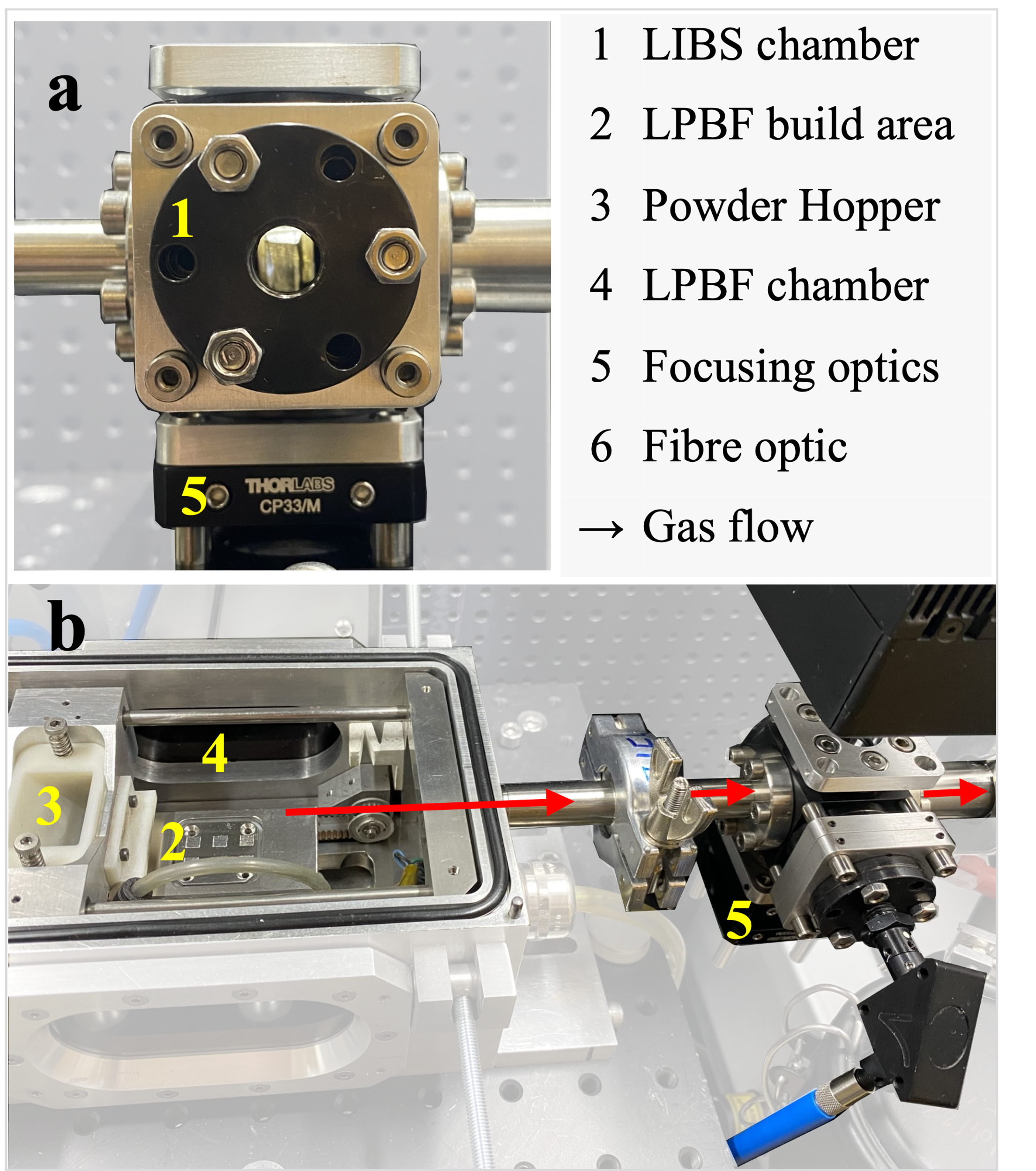


### Supplementary Figure 5: The apparatus used to sample solid-state and vapour-state IN625 to generate the DCSP plots and perform large print LPBF

**(a)** LIBS was performed on the feedstock IN625 substrate in the centre of the LIBS chamber to generate the DCSP calibration plots for quantitative analysis of LIBS data. **(b)** A picture showing the arrangement of the LIBS system relative to the LPBF build area.


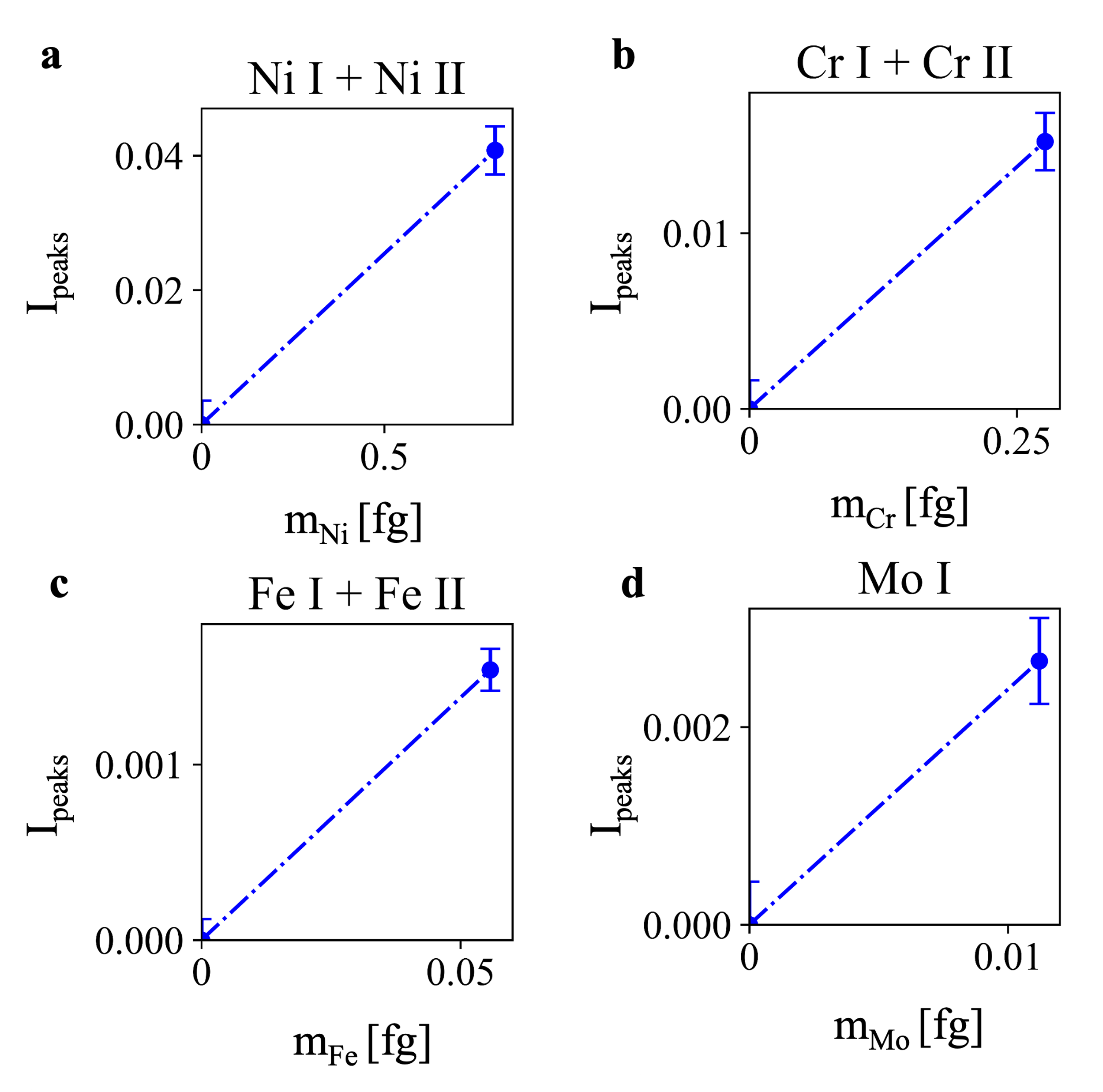


### Supplementary Figure 6: DCSP plots for each of the main alloying constituents of IN625

The plots demonstrate the linear regression fitting of sampled mass against summed peak intensities, for **(a)** Ni, **(b)** Cr, **(c)** Fe and **(d)** Mo. The mass units are given in femtograms [fg].


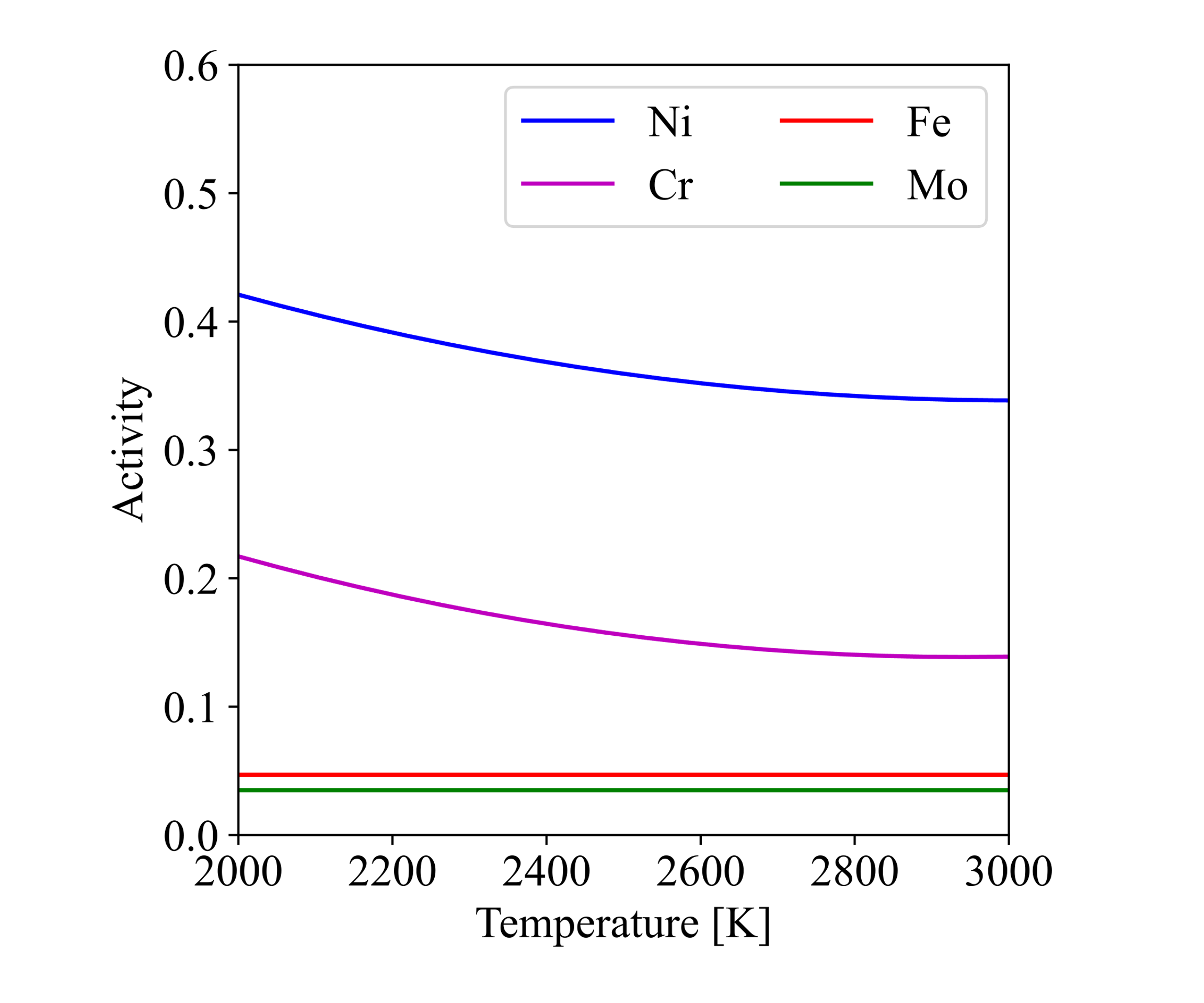


### Supplementary Figure 7: Temperature-dependent activities used in the simulations

The activities were provided by the Thermo-Calc software database.^2^ The total activity equals the elemental activity coefficient multiplied by the mole fraction for each element.


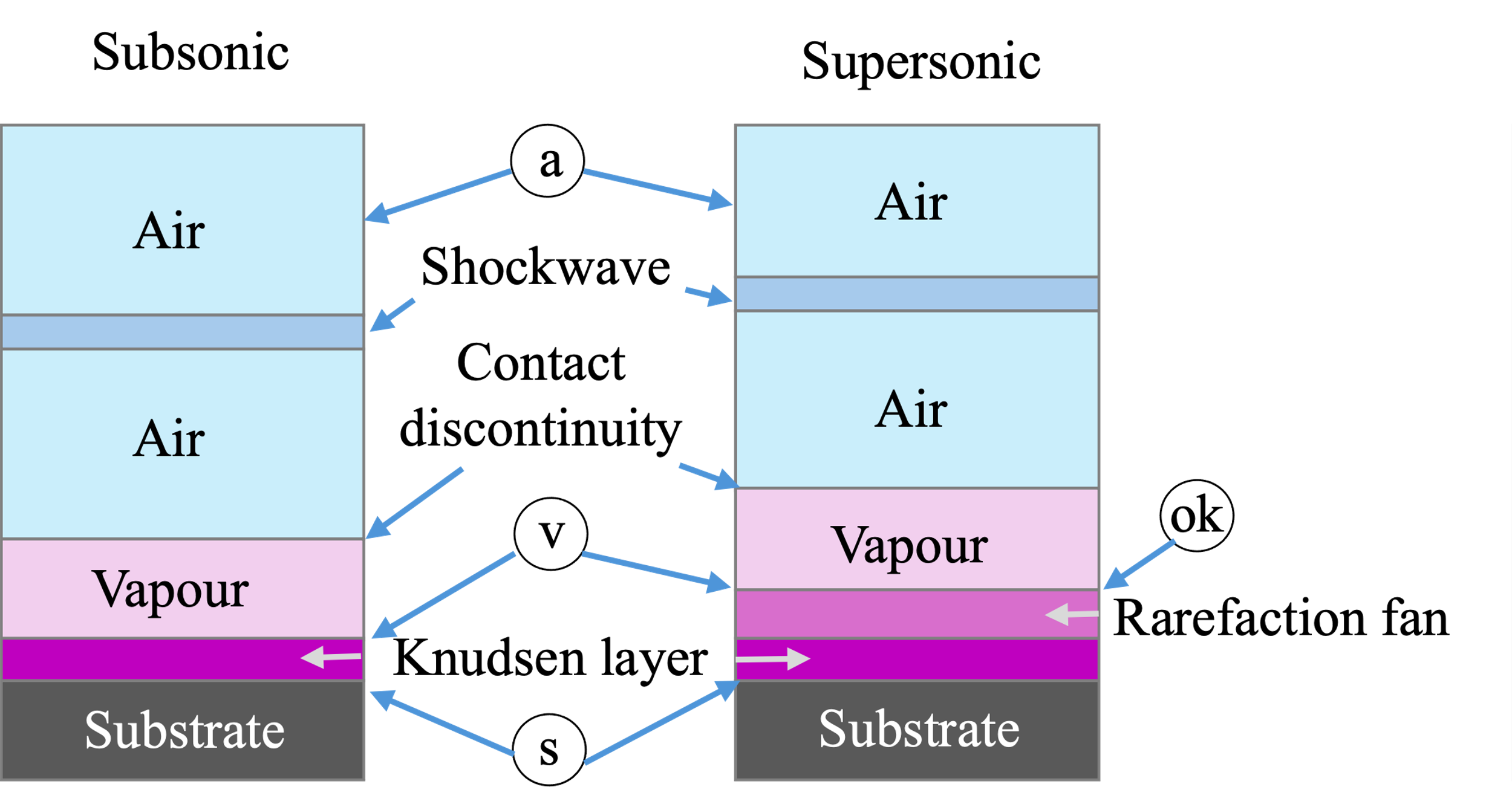


### Supplementary Figure 8: Schematic of the Knudsen – vapour layer approximation used in the Ki *et al.* model after ^3^

Similar assumptions were also employed by Wang *et al*.^4^ The circled letters are subscripts used in **Equations (12-19)** in the main text, and **Equations (S18-S20)** in the **Supplementary Information**.


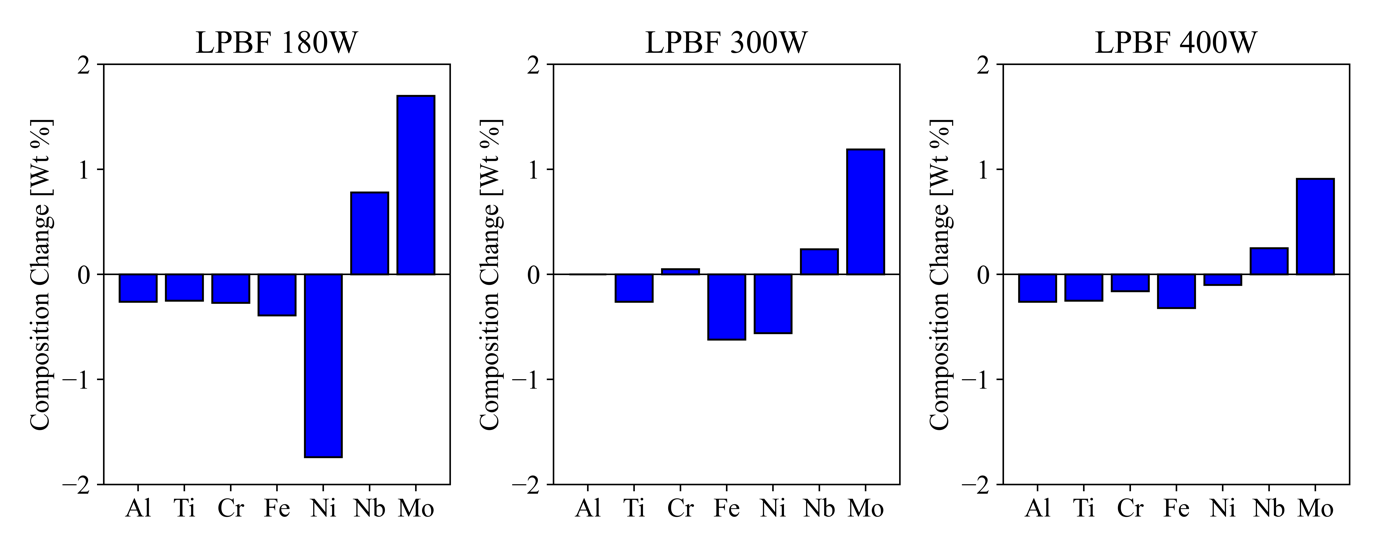


Supplementary Figure 9: SEM-EDS results showing the change in composition after LPBF printing over a large print area

For 180 W, 300 W, and 400 W laser powers. To varying degrees, we quantify compositional reduction of Al, Ti, Cr, Fe and Ni – and compositional percentage gains of Nb and Mo, and occasionally Cr (at 300 W). In the main text analysis, the trace elements are omitted for clarity. The normalisation process causes a net percentage increase in Cr due to the omission of Nb and trace elements in the calculation, representing that Ni and Fe are typically lost at greater rates than Cr.


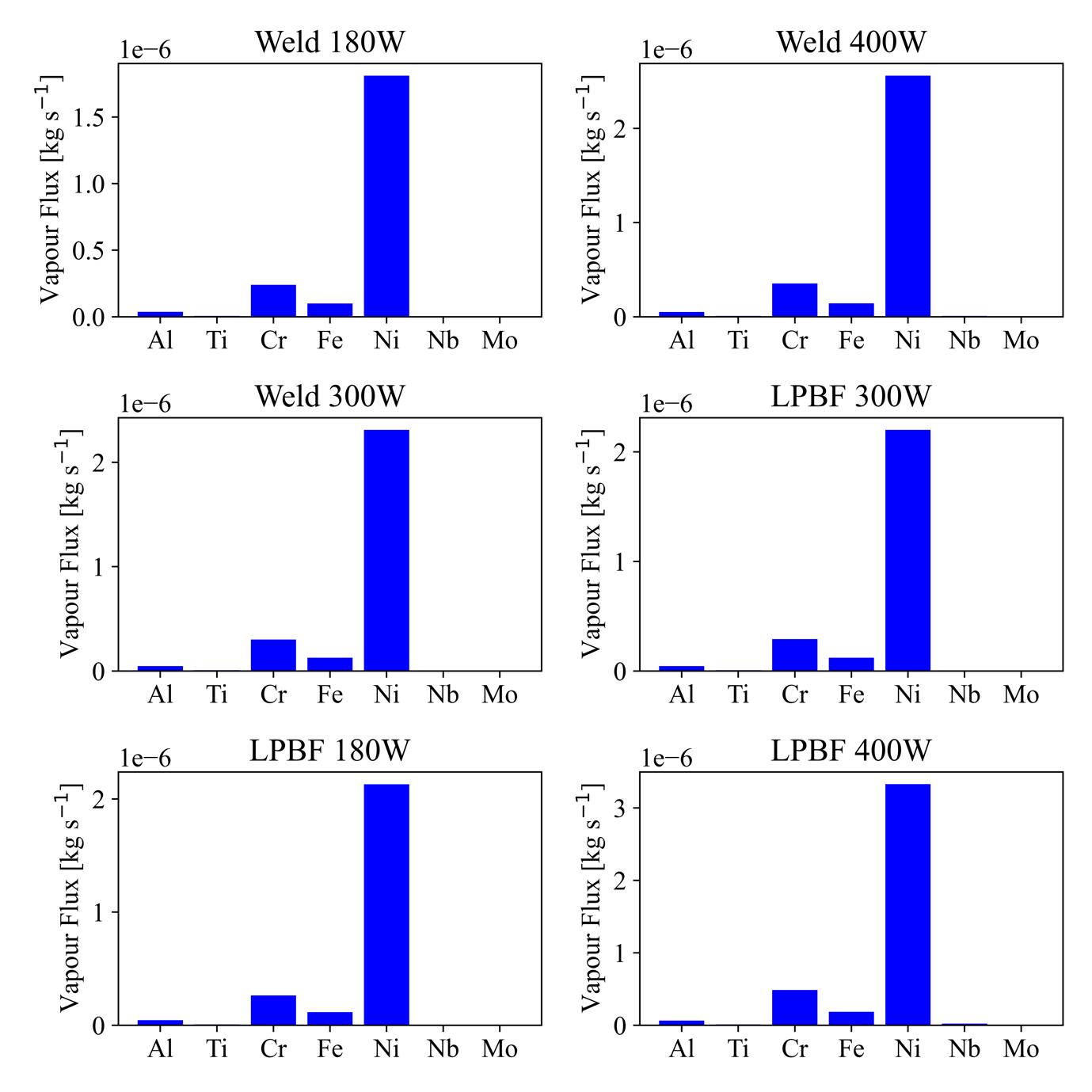


Supplementary Figure 10: The vapour flux emitted for all elements, predicted by the Flint *et al.* model. ^1^

In the main text, the trace elements are omitted to allow better clarity compared to the LIBS results. We observe a large presence of Ni, with some Cr and Fe, and trace amount of Al.

## Supplementary Tables

### Supplementary Table 1: *Ex situ* bulk compositions of IN625 substrate and powder materials

The chemical compositions were measured by calibrated Scanning Electron Microscopy Energy Dispersive X-ray Spectroscopy (SEM-EDS) - see **Supplementary Methods 1**. The compositions have been normalised to 100% to negate the effect of trace elements in the study. The normalised nominal composition of the substrate (Goodfellow, UK) is 62% Ni, 23% Cr, 10% Mo, 5% Fe.

| **Sample** | **Ni [Wt %]** | **Cr [Wt %]** | **Fe [Wt %]** | **Mo [Wt %]** |
| --- | --- | --- | --- | --- |
| **Powder** | 66.65 *± 0.78* | 22.35 *± 0.45* | 2.88 *± 0.24* | 8.12 *± 0.63* |
| **Substrate** | 65.89 *± 0.48* | 21.31 *± 0.27* | 4.31 *± 0.18* | 8.49 *± 0.39* |

The raw, unnormalized, chemical compositions of the IN625 substrate and powder materials – measured by SEM-EDS - are provided in the table below. For the powder sample, 5.37 wt% oxygen was detected.

| **Sample** | **Ni** | **Cr** | **Fe** | **Mo** | **Nb** | **Ti** | **Al** | **Mn** | **V** |
| --- | --- | --- | --- | --- | --- | --- | --- | --- | --- |
| **Powder** | 58.84 | 21.00 | 2.76 | 7.79 | 3.35 | 0.41 | 0.25 | 0.18 | 0 |
| **Substrate** | 61.81 | 21.28 | 4.29 | 8.66 | 3.46 | 0.24 | 0.25 | 0 | 0.06 |

### Supplementary Table 2: Vapour depression surface area measurements from the segmented radiographs

The vapour depression surface area (σ) was approximated as the surface area of a circular cone using $\sigma= {\pi AD}/2$ where A is the vapour depression aperture width and D is the vapour depression depth.

| **Metric** | **Welding Conduction** | **Welding Keyhole** | **LPBF Conduction** | **LPBF**  **Keyhole** |
| --- | --- | --- | --- | --- |
| **Vapour depression surface area (µm^2^)** | 1.10 x 10^4^  *± 0.09* | 4.82 x 10^4^  *± 0.12* | 1.07 x 10^4^  *± 0.09* | 3.23 x 10^4^ *± 0.05* |
| **Vapour depression depth (µm)** | 55 *± 10.5* | 277 *± 17.6* | 95 *± 9.5* | 302 *± 18.2* |

### Supplementary Table 3: Numerical simulation of the melt pool and keyhole characteristics

The numerical simulation was performed using the Flint *et al*.^1^ modelling approach. All results are taken at time, *t* = 500 µs, when the simulation had reached steady-state conditions.

| **Metric** | **Welding Conduction** | **Welding Keyhole** | **LPBF Conduction** | **LPBF**  **Keyhole** |
| --- | --- | --- | --- | --- |
| **Peak temperature (K)** | 3386 | 3386 | 3386 | 3386 |
| **Melt pool volume (µm^3^)** | 0.92 x 10^6^ | 1.81 x 10^6^ | 0.94 x 10^6^ | 2.08 x 10^6^ |
| **Average melt pool temperature (K)** | 2364 | 2334 | 2282 | 2312 |
| **Melt pool surface area (µm^2^)** | 1.41 x 10^4^ | 2.63 x 10^4^ | 1.76 x 10^4^ | 3.10 x 10^4^ |
| **Average melt pool surface temperature (K)** | 2836 | 2792 | 2704 | 2754 |
| **Vapour depression surface area (µm^2^)** | 1.2 x 10^4^ | 2.4 x 10^4^ | 1.6 x 10^4^ | 2.9 x 10^4^ |
| **Vapour depression depth (µm)** | 48 ± 8 | 100 ± 20 | 33 ± 13 | 110 ± 20 |
| **Total vaporisation surface Area (µm^2^)** | 2.61 x 10^4^ | 5.03 x 10^4^ | 3.36 x 10^4^ | 6.10 x 10^4^ |

The average melt pool temperature is given by:

|  | $T_{pool, avg}= \frac{1}{V_{pool}}\int_{pool} T dV_{pool}$ | Equation (S1) |
| --- | --- | --- |

where the temperature $T$ is integrated over the pool volume, $V_{pool}$.

The average melt pool surface temperature is given by:

|  | $T_{surf, avg}= \frac{1}{\sigma_{melt}}\int_{pool} T d\sigma_{melt}$ | Equation (S2) |
| --- | --- | --- |

where the temperature is averaged over the melt pool surface area, $\sigma_{melt}.$

Under higher laser power, we observe a larger melt pool due to the increased energy input. However, the peak temperature reached in both cases is similar – *i.e.,* close to the boiling point; due to the increased energy losses through vaporisation in high power cases inferring a self-limitation on melt temperature. Therefore, larger average melt temperatures are seen in the conduction case, owing to the smaller pool, yet the same peak temperature. The total vaporisation surface area, calculated by summing the melt surface area and vapour depression surface area, is given in **Supplementary Table 3**. From conduction to keyhole mode, the total surface area nearly doubles in each case (a 93 % increase under welding conditions, and 82 % increase under LPBF). This increase in surface area allows for a stronger vaporisation response at higher laser powers, despite the temperature of the bulk liquid remaining similar.

Supplementary Table 4: Vapour compositions measured during welding-LIBS and LPBF-LIBS in the laboratory and synchrotron experiments

The error bars represent the standard error, $SE= \sigma/\sqrt{n}$ , where $\sigma$ is the standard deviation and $n$ is the number of spectra measured over three layers. Note the change of unit: femtograms [fg] in the synchrotron results, and picograms [pg] for the large print results.

| **Experiment** | **Power [W]** | **Ni [fg]** | **Cr [fg]** | **Fe [fg]** | **Mo [fg]** |
| --- | --- | --- | --- | --- | --- |
| **Welding (synchrotron)** | 180 | 20570 *± 765* | 3802 *± 321* | 1950 *± 103* | 1180 *± 100* |
|  | 400 | 88400 *± 1360* | 18600 *± 711* | 6280 *± 240* | 1890 *± 154* |
| **LPBF (synchrotron)** | 180 | 394 *± 13.3* | 73.7 *± 3.4* | 28.7 *± 3.9* | 4.46 *± 1.7* |
|  | 400 | 368 *± 9.82* | 63.2 *± 4.0* | 33.5 *± 19.4* | 10.1 *± 1.3* |
| **Experiment** | **Power [W]** | **Ni [pg]** | **Cr [pg]** | **Fe [pg]** | **Mo [pg]** |
| **LPBF**  **(large print)** | 180 | 814 *± 52* | 177 *± 25* | 56.9 *± 6.6* | 3.1 *± 1.8* |
|  | 300 | 835 *± 57* | 146 *± 10* | 52.6 *± 11* | 29.9 *± 0.9* |
|  | 400 | 1070 *± 55* | 205 *± 5.2* | 51.6 *± 5.3* | 5.8 *± 3.6* |

### Supplementary Table 5: LIBS loss ratio results from the synchrotron experiments

The LIBS loss ratios were calculated using **Equation (3)** in the main text.

| **Experiment** | **Power [W]** | **Ni** | **Cr** | **Fe** | **Mo** |
| --- | --- | --- | --- | --- | --- |
| **Welding (synchrotron)** | 180 | 0.295 | 0.168 | 0.430 | 0.107 |
|  | 400 | 0.348 | 0.226 | 0.379 | 0.047 |
| **LPBF (synchrotron)** | 180 | 0.361 | 0.208 | 0.404 | 0.026 |
|  | 400 | 0.322 | 0.171 | 0.451 | 0.056 |
| **LPBF**  **(large print)** | 180 | 0.361 | 0.243 | 0.388 | 0.009 |
|  | 300 | 0.365 | 0.198 | 0.354 | 0.082 |
|  | 400 | 0.423 | 0.250 | 0.313 | 0.014 |

### Supplementary Table 6: Vapour compositions estimated by three simulation approaches

The mass unit is nanograms [ng].

| **Experiment** | **Power [W]** | **Ni [ng]** | **Cr [ng]** | **Fe [ng]** | **Mo [ng]** |
| --- | --- | --- | --- | --- | --- |
| **Welding**  **(Flint *et al.*)** | 180 | 29000 | 3850 | 1590 | 246 |
|  | 300 | 36900 | 4810 | 2020 | 392 |
|  | 400 | 41000 | 5660 | 2270 | 159 |
| **LPBF**  **(Flint *et al.*)** | 180 | 34100 | 4200 | 1860 | 582 |
|  | 300 | 35100 | 4660 | 1930 | 302 |
|  | 400 | 53300 | 7780 | 2970 | 163 |
| **Welding**  **(Ki *et al*./Wang *et al*.)** | 180 | 15000 | 11600 | 1210 | 4.11 |
|  | 300 | 18000 | 13700 | 1440 | 6.04 |
|  | 400 | 24000 | 17900 | 1900 | 6.70 |
| **LPBF**  **(Ki *et al*./Wang *et al*.)** | 180 | 17800 | 13600 | 1420 | 4.58 |
|  | 300 | 21400 | 16000 | 1700 | 6.49 |
|  | 400 | 33300 | 25600 | 2680 | 11.6 |
| **Welding (Langmuir)** | 180 | 3920 | 3010 | 525 | 2.50 |
|  | 300 | 2380 | 2840 | 747 | 3.83 |
|  | 400 | 4610 | 3090 | 781 | 3.84 |
| **LPBF**  **(Langmuir)** | 180 | 3700 | 2420 | 627 | 3.15 |
|  | 300 | 3850 | 2470 | 659 | 3.44 |
|  | 400 | 7240 | 4710 | 1230 | 6.34 |

### Supplementary Table 7: Loss ratio results from three simulation approaches

The model loss ratios were calculated using **Equation (3)** in the main text.

| **Experiment** | **Power [W]** | **Ni** | **Cr** | **Fe** | **Mo** |
| --- | --- | --- | --- | --- | --- |
| **Welding**  **(Flint *et al*.)** | 180 | 0.433 | 0.177 | 0.366 | 0.024 |
|  | 300 | 0.432 | 0.174 | 0.365 | 0.024 |
|  | 400 | 0.434 | 0.185 | 0.370 | 0.011 |
| **LPBF**  **(Flint *et al.*)** | 180 | 0.430 | 0.164 | 0.360 | 0.046 |
|  | 300 | 0.433 | 0.177 | 0.366 | 0.024 |
|  | 400 | 0.430 | 0.194 | 0.369 | 0.008 |
| **Welding**  **(Ki *et al*./Wang *et al*.)** | 180 | 0.216 | 0.516 | 0.267 | 0.00004 |
|  | 300 | 0.221 | 0.510 | 0.269 | 0.00004 |
|  | 400 | 0.221 | 0.510 | 0.269 | 0.00004 |
| **LPBF**  **(Ki *et al*./Wang *et al*.)** | 180 | 0.218 | 0.514 | 0.268 | 0.00004 |
|  | 300 | 0.219 | 0.513 | 0.269 | 0.00005 |
|  | 400 | 0.217 | 0.515 | 0.268 | 0.00005 |
| **Welding (Langmuir)** | 180 | 0.184 | 0.437 | 0.379 | 0.00007 |
|  | 300 | 0.178 | 0.356 | 0.466 | 0.0001 |
|  | 400 | 0.176 | 0.365 | 0.459 | 0.00009 |
| **LPBF**  **(Langmuir)** | 180 | 0.178 | 0.359 | 0.463 | 0.0001 |
|  | 300 | 0.178 | 0.353 | 0.468 | 0.0001 |
|  | 400 | 0.178 | 0.356 | 0.466 | 0.0001 |

### Supplementary Table 8: *Ex situ* bulk compositions of the large area builds

The cross-sections of the samples were mapped by calibrated EDS, and then normalised to 100% to negate the effect of trace elements.

| **Sample** | **Ni [Wt %]** | **Cr [Wt %]** | **Fe [Wt %]** | **Mo [Wt %]** |
| --- | --- | --- | --- | --- |
| **180 W** | 63.62 *± 0.12* | 22.49 *± 0.07* | 3.58 *± 0.04* | 10.30 *± 0.11* |
| **300 W** | 64.20 *± 0.11* | 22.51 *± 0.06* | 3.63 *± 0.03* | 9.65 *± 0.1* |
| **400W** | 64.62 *± 0.10* | 22.45 *± 0.06* | 3.35 *± 0.03* | 9.58 *± 0.09* |

### Supplementary Table 9: LIBS calibration coefficients from the DCSP method

The plots utilise a linear interpolation of form $I_{peaks}=n\left( m_{i} \right)+c$ for element *i* (see **Methods**).

| **Fit Result** | **Ni** | **Cr** | **Fe** | **Mo** |
| --- | --- | --- | --- | --- |
| ***n*** | 5.09 x10^10^ | 5.51 x10^10^ | 2.77 x10^10^ | 2.38 x10^10^ |
| ***c*** | 0 | 0 | 0 | 0 |

### Supplementary Table 10: EDS calibration coefficients

The coefficients were extracted using a linear interpolation $y = mx + c$, where $y$ is the composition measured by EDS, and $x$ is the SRM-calibrated composition - see **Supplementary Methods 1**.

| **EDS Fit Result** | **Ni** | **Cr** | **Fe** | **Mo** |
| --- | --- | --- | --- | --- |
| ***m*** | 0.979 | 1.043 | 1.0012 | 1.065 |
| ***c*** | 0.000545 | -0.0226 | 0.152 | -0.0115 |

### Supplementary Table 11: Melt pool temperature percentage differences from the vapour pressure interpretation

The percentage difference between the Ni and Fe LIBS loss ratios, which is used to determine the percentage melt pool temperature difference - see **Supplementary Discussion 3**.

| **Vapour pressure** | **Type** | **Conduction** | **Keyhole** | **Average** |
| --- | --- | --- | --- | --- |
| **% diff.** | Weld | 13.5 % | 0.3 % | 6.2 % |
|  | LPBF | 4.3 % | 12.9 % | 8.6 % |

## Supplementary Discussions

### Supplementary Discussion 1: Disparity between simulation and experimental keyhole depths

The keyhole depth for the 400 W case is shallower than the experimental depth, with strong temporal depth fluctuations. This may be attributed to the uncertainty in the local laser absorptivity; as this is difficult to measure, we use a constant value of 0.6. Another possible reason for the disparity is our assumption that cold powders would not move in this simulation, resulting in a partial heat transfer to powders in the melt pool periphery. Given that the total input laser energy is the same as the experimental one, the partial heat transfer (*i.e.,* the rise in temperature) to the periphery powders would cause a slightly larger redistribution of recoil pressure at the keyhole entrance (resulting in a larger keyhole entrance diameter). Nonetheless, the melt pool surface area is not overly affected, and the vaporisation behaviour is similar to the experimental trend. The loss ratio analysis further minimises the effect of these uncertainties when comparing between the cases.

To identify the associated error, we use the welding synchrotron X-ray radiography case, as this experiment has better LIBS coupling (see **Supplementary Discussion 2**), alongside vapour depression measurements. In the welding experiment, we observe a 320 % increase in vapour mass and a 400 % increase in keyhole depth when the melting mode switches from conduction to keyhole mode. Therefore, the vapour mass increases by a factor of 0.8 relative to the keyhole depth.

In the simulation results, we observe a vapour mass increase of 42 %, alongside a keyhole depth increase of 108 %. Hence, the vapour mass scales by a factor of 0.4 relative to keyhole depth. If the simulation keyhole depth were to be more in-line with experimental work, we may expect the increased amount of laser reflections within the keyhole to close this gap, potentially increasing the predicted vapour mass to double the predicted value; for example, predicting 69,400 ng for the keyhole welding, compared to the current 49,100 ng prediction.

### Supplementary Discussion 2: Synchrotron X-ray radiography error margins

Possible errors in synchrotron X-ray radiography experiments may include: (i) changes in composition due to the mixing of powder feedstock and substrate materials during LPBF and (ii) a limited scan area, due to spatial restrictions employed to ensure adequate X-ray beam transmission through IN625, resulting in a smaller vapour plume for LIBS sampling. Hence, the measurements are much more susceptible to fluctuations – *e.g.,* events of strong laser coupling to a particulate in the vapour. This effect was less obvious in the welding experiment, as we observed the weld vapour to couple more efficiently with the LIBS system. This is because the welding plume contains mostly vaporised species - as previously revealed by Schlieren imaging.^5^ By comparison, the LPBF plume is much more complex, and emitted vapours may condense into liquid and powder spatter, further reducing the sample volume within the LIBS system under low plume loads.^6^

### Supplementary Discussion 3: Vapour pressure calculation of melt pool temperature difference and vapour condensation discussion

In the main text, we estimated the temperature differences between welding and LPBF melt pools using the vapour pressure plots. We estimated the percentage difference between Ni and Fe loss ratios for each condition, and determined the difference (% diff.) as:

|  | $\% diff. = \frac{Fe loss ratio-Ni loss ratio}{Total loss ratio} x 100$ | Equation (S3) |
| --- | --- | --- |

where the total loss ratio equals 1. Given that the melt pool temperature is correlated to the vapour pressure plots, we estimate that the percentage change in melt pool temperature is linked to the percentage difference of the Ni and Fe vapour pressure curves. We expect an average 6.2 % difference between the Fe and Ni vapour pressure curves under welding conditions; this difference occurs at a temperature T_1_ on the vapour pressure plot. Under LPBF, we expect the average 8.6 % difference to occur at a temperature T_2_ on the vapour pressure plot. The average temperature difference between welding and LPBF (T_1_ – T_2_) ~ 8.6% - 6.2% = 2.4 % - see details in **Supplementary Table 11**.

Similarly, the percentage melt pool temperature difference between welding (T_weld_) and LPBF (T_LPBF_), was calculated as:

|  | $\% T diff. = \frac{T_{weld}- T_{LPBF}}{T_{weld}} x 100$ | Equation (S4) |
| --- | --- | --- |

Using this equation, we find a simulated temperature difference ($\% T diff.$) between welding and LPBF of 3.5% in conduction mode, and 0.9% in keyhole mode. Therefore, the average temperature difference between welding and LPBF in the Flint *et al.* simulation is 2.2%.

The reduced melt pool temperatures in LPBF, compared to welding, could be attributed to a higher condensation rate due to vapour condensation onto the cold powder layer and spatter within the plume. The vapour detected by the LIBS system, and the simulated evaporative losses, are a function of the net vaporisation (J_net_ = J_m_ - φ_m_ for evaporation flux, J_m_, and condensation rate, φ_m_). A large proportion of the initial vaporisation occurs at the laser-metal interaction zone, but condensation can occur at any point along the liquid-gas surface, depending on the local pressure. We hypothesise that the loss ratios indicate the average melt pool temperature rather than the peak temperatures.

### Supplementary Discussion 4: Large print area LPBF-LIBS

From the LPBF-LIBS results over a large printing area, we observe an anomaly in the trend of Cr loss (**Supplementary Table 4**). The vaporised mass of Cr during 300 W LPBF is lower than expected, compared to 180 W and 400 W, in turn leading to a drop in the normalised loss ratio. However, the EDS post-build measurements show a slight wt.% increase in Cr, indicating a reduced amount of Cr in the vapour under 300W LPBF conditions (**Supplementary Figure 9**). The Flint *et al.* ^1^ simulation shows that the 300 W case is in the transition melting mode; there may be an interim vapour behaviour not being captured in the current study. Further work, across a wider range of conditions, is needed to understand this deviation.

### Supplementary Discussion 5: Behaviour of Nb during LPBF:

Throughout the main text, we refer to the main alloying constituents of IN625 as Ni, Cr, Fe and Mo. As shown in **Supplementary Table 1**, there is a significant Nb presence in the IN625 alloy. The DCSP method relies on database information provided by the NIST LIBS database – however, Nb is not included in this database.^7^ Therefore, the DCSP plot could not be reliably created and we omitted Nb from the LIBS results.

If Nb were to be included, we would expect similar behaviour to Mo within the IN625 vapour, due to the high boiling point of Nb (5017 K). Nb would not be prone to vaporisation, hence we would expect minimal vapour presence and an overall increase in the Nb wt% of IN625 after welding and LPBF. These expectations are reflected in both the full SEM-EDS results (see **Supplementary Fig. 9**) and the Flint *et al.* simulation (see **Supplementary Fig. 10**).^1^ The former shows net increases of Nb in the post-LPBF builds; of 0.78% at 180 W, 0.24 wt% at 300 W, and 0.25 wt% at 400 W. The latter shows very minimal Nb predicted in the IN625 vapours by simulation, between 0 – 2.25 x 10^-8^ kg s^-1^. It is therefore reasonable to assume very little Nb vaporisation during the welding and LPBF of IN625.

### Supplementary Discussion 6: LIBS calibration methods

Quantitative analysis using LIBS requires careful calibration, which can be challenging; the technique suffers from matrix effects (signal behaviour is dependent upon chemical and physical properties of the plasma), self-absorption of spectral lines and plasma intensity variations that are difficult to control. ^8^ Further to this, there is significant spectral overlap and background phenomena – hence complex processing and deconvolution techniques are required. ^9^ Once deconvolution has been performed, usually by curve-fitting or deconvolution algorithms, ^10–13^ quantitative analysis can proceed, however, this approach must consider matrix effects and experimental factors.

Methods to cope with such problems can typically be categorised into one of two approaches: calibration curves or ‘calibration-free’ methods. The calibration curve method involves performing LIBS upon a series of known samples, with a similar composition to the unknown sample, with gradually increasing concentrations of the analyte of interest. ^14,15^ Plots of the LIBS intensity against sample concentrations will be created, and the unknown sample will be compared against the plot fit to infer the concentration of the unknown analyte.

By contrast, the ‘calibration-free’ method is a theoretical approach, by which the concentration is calculated using the Boltzmann equation (or, variations of the Boltzmann equation, such as the Saha-Boltzmann equation). The Boltzmann equation states:

|  | $I= FC_{s}A_{ki}\frac{g_{k}e^{\frac{{-E}_{k}}{k_{\beta}T}}}{U_{s}(T)}$ | Equation **(**S5**)** |
| --- | --- | --- |

Where *I* is the peak intensity, *F* is the wavelength-dependent spectrometer efficiency, $C_{s}$ is the species concentration (e.g., Ni I – for elemental concentration, this equation must be solved for each ionised species of a given element - typically the I and II state), $A_{ki}$ is the transition probability, $g_{k}$ is the upper level degeneracy, $E_{k}$ is the upper energy level, $k_{\beta}$ is the Boltzmann constant, $T$ is the plasma temperature and $U_{s}(T)$ is the temperature-dependent partition function. The Boltzmann equation can be rearranged as:

|  | $\ln\left( \frac{IA_{ki}}{g_{k}} \right)=-\frac{E_{k}}{k_{\beta}T}+\ln\left( \frac{FC_{s}}{U_{s}\left( T \right)} \right)$ | Equation (S6) |
| --- | --- | --- |

Which can be plotted as a linear relation, with the terms:

$$y=\ln\left( \frac{I}{A_{ki}g_{k}} \right), m= -\frac{1}{K_{\beta}T} , x= E_{k}, c=\ln\left( \frac{C_{s}F}{U_{s}\left( T \right)} \right)$$

As $A_{ki}$ and $g_{k}$ can be extracted from the NIST LIBS database^7^, we can produce a linear regression plot, with slope inversely proportional to *T* and the species concentration proportional to the y-intercept. The species concentration can be extracted from the y-intercept, c, using a closure relation:

|  | $\sum_{s} C_{s}=\frac{1}{F}\sum_{s} U_{s}\left( T \right)\exp\left( c \right)=1$ | Equation (S7) |
| --- | --- | --- |

Hence quantitative results can be extracted. However, this method relies on having a known value of *F* – which was not known in this study. There is a variation of ‘calibration-free’ LIBS by which the *F* parameter can be deduced from a measurement of a single known composition; called ‘One-Point Calibration’ (OPC) LIBS.^16^ This is achieved by calculating the difference between the theoretical intensity of a peak of known composition using the Boltzmann equation, and the measured peak intensity from the experimental apparatus. The equations are nicely described by Hu *et al.* ^17^ (**Equations 1 to 6**). The OPC method is suitable for metal alloy systems; traditional calibration curves are not appropriate, due to the difficulty of obtaining suitable Standard Reference Materials (SRMs) for metal alloys.

For this study, we performed OPC analysis using the feedstock material as the known composition (measured using SRM-calibrated EDS). However, our system required the ability to extract quantitative results from vapour LIBS using solid-state calibration materials. To achieve this, we proposed a hybrid technique; we performed LIBS upon the solid-state feedstock material, before using OPC analysis from the known composition to calculate the plasma density ($C_{s}$) and plasma temperature from the solid-state LIBS experiment. The mass of alloy sampled in the LIBS plasma was calculated using the plasma density and an estimation of the plasma volume. As detailed in the **Methods**, the calculated mass was used to construct Density-Corrected Single-Point (DCSP) plots for quantitative analysis.

The assumption in our hybrid DCSP approach is that the vaporised masses cannot exceed the sampled solid-state masses, as the solid will always more dense than the vapour phase. We also assume a linear relationship of peak intensity to mass; the small sample size means it would be overly assumptive to adopt any other relationship. This technique enables us to determine the metal vapour composition relative to its feedstock alloy – and as such, the results are best interpreted as a comparison of vapour compositions rather than as absolute vaporisation mass values.

### Supplementary Discussion 7: Preferential vapour condensation

Throughout this work, we consider the preferential vaporisation effect due to varying vapour pressures of the constituent elements in IN625. This is measured by sampling the vapour plume using LIBS at the exhaust of the LPBF chamber. The differing vapour pressures of Fe, Cr, Ni and Mo have two-fold implications; the elements with the highest vapour pressures will, firstly, be vaporised at greater rates – and secondarily, will condense at greater rates, as the condensation rate of a given element can be considered proportional to its vapour pressure, as per the equation:^18^

|  | $J_{net}=J_{m}- J_{con}= \Phi J_{m}$ | Equation (S8) |
| --- | --- | --- |

where $J_{net}$ is the practical vaporisation flux, $J_{m}$ is the vaporisation flux calculated using the Langmuir equation, and $\Phi$ is a factor dependent on the condensation flux $J_{con}$. Therefore, as the vapour travels towards the LIBS chamber, we may expect preferential condensation of the elements with the highest vapour pressures (Fe, Cr and Ni) along the exhaust walls, which may affect the LIBS-measured results. Here, we provide an analysis of the error induced from the preferential condensation along the exhaust walls.

We assume the exhaust wall condensation is predominately within the Knudsen layer of the metal wall. The mean free path, $\lambda$, for the system may be described as: ^19^

|  | $\lambda\sim\frac{1}{\sqrt{2} n\pi d}$ | Equation (S9) |
| --- | --- | --- |

For $n$ particles with cross-sectional area $d.$ Assuming the Knudsen layer is ~ 3 times the mean free path, and substituting $n=p/k_{b}T$, we can represent the Knudsen layer thickness, $l_{c}$, as:

|  | $l_{c}\sim\frac{3kT}{\sqrt{2}\pi d^{2}p}$ | Equation (S10) |
| --- | --- | --- |

where $k$ is the Boltzmann constant, $T$ is the temperature, $d$ is the molecular diameter, and $p$ is the pressure. Given that the exhaust gases are > 99.9 % Ar, we take the molecular diameter of argon, $d$ = 340 pm; the pressure is taken to be the same as the chamber pressure, $p$ = 101 kPa. We can consider the temperature range of ambient ($T$ = 298 K) to IN625 boiling temperature of IN625 ($T$ ~ 3250 K). These values give a Knudsen layer thickness ranging between 2.38 x 10^-7^ m and 2.59 x 10^-6^ m.

We will take the worst-case scenario by assuming $l_{c}$ = 2.59 x 10^-6^ m, and a condensation rate equal to the vaporisation rate ($J_{\mathrm{con}}$ = $J_{m}$). The inner diameter of the exhaust is 10 mm – therefore, the fraction of argon gas and metal vapour flowing through the exhaust that is contained within the Knudsen layer is:

$$A_{l_{c}}=\left( \pi\left( 5 \right)^{2}-\pi\left( 5-0.00259 \right)^{2} \right)=25\pi-24.97\pi=0.03\pi$$

Therefore, ~ 0.12 % of the vapour is contained within the Knudsen layer – of which the majority is argon shielding gas. Extrapolating from the vapour pressure equations given in **Methods**; the metal vapour condensate would be composed of ~ 0.12% Fe at 298 K relative to the total vapour composition; and composed of 0.06% Ni, 0.06% Fe and 0.006% Cr relative to the total metal vapour composition at 3250 K. It can therefore be assumed that any preferential condensation effects will have a negligible effect on the LIBS vapour sampling results.

## Supplementary Methods

### Supplementary Methods 1: Energy Dispersive X-ray Spectroscopy calibration

To construct the Scanning Electron Microscope Energy Dispersive X-ray Spectroscopy (SEM-EDS) calibration curves, measurements of four alloy (NiCoCr, Ti-6Al-4V, SS-316L, Al-380) Standard Reference Materials (SRMs) from the National Institute of Standards and Technology (NIST) were sampled by EDS. The calibration curves were constructed as a linear regression of the form $y = mx + c$, where $y$ is the composition as measured by EDS, and $x$ is the SRM calibrated composition. The curves included a standard error ($SE= \sigma/\sqrt{n}$ where $\sigma$ is the standard deviation and $n$ is the sample size) for each point. The fitting coefficients are given in **Supplementary Table 10**. To ensure chemical composition accuracy, all EDS results were re-scaled according to these calibration coefficients.

### Supplementary Methods 2: Simulation Methodology

The following section details the multi-physics modelling methodology, based on computational fluid dynamics (CFD).^20,21^ The fluid flow equations of mass, momentum and temperature were solved in a fully coupled way, given as:

|  | $\frac{\partial\rho}{\partial t}+(\vec{\boldsymbol{u}}\cdot\nabla)\rho=-\rho\nabla\cdot\vec{\boldsymbol{u}}$ | Equation (S11) |
| --- | --- | --- |
|  | $\frac{\partial u}{\partial t}+(\vec{\boldsymbol{u}}\cdot\nabla)\vec{u}=-\frac{\nabla p}{\rho}+\vec{\boldsymbol{Q}_{\boldsymbol{u}}}+\vec{\boldsymbol{g}}+\vec{{\frac{\boldsymbol{1}}{\boldsymbol{\rho}}\boldsymbol{F}}_{\boldsymbol{u,surf}}}$ | Equation (S12) |
|  | $\frac{\partial T}{\partial t}+(\vec{\boldsymbol{u}}\cdot\nabla)T=-\frac{p\nabla\cdot\vec{\boldsymbol{u}}}{\rho c_{p}}+Q_{T}$ | Equation (S13) |

where $\rho$ is the density, $\vec{\boldsymbol{u}}$ is the velocity, *T* is the temperature, *p* is the pressure and *c_p_* is the constant-pressure heat capacity. $\vec{\boldsymbol{Q}_{\boldsymbol{u}}}$represents the viscous effects including the Newtonian viscous force and Darcy’s force in the mushy zone, which is given by:

|  | $Q_{u,i}=\frac{1}{\rho}\frac{\partial\tau_{ij}}{\partial x_{j}}-\frac{\nu}{K}\left( \frac{(1-f_{L})^{2}}{{f_{L}}^{3}} \right)u_{i},\quad\tau_{ij}=\mu\left( \frac{\partial u_{i}}{\partial x_{j}}+\frac{\partial u_{j}}{\partial x_{i}} \right)-\frac{2}{3}\mu\nabla\cdot\vec{\boldsymbol{u}}\delta_{ij}$ | Equation (S14) |
| --- | --- | --- |

where *K* is the permeability coefficient, *f_L_* is the liquid fraction determined by the temperature, $\nu$ is the kinematic viscosity and $\mu$ is the dynamic viscosity. ***g*** is the gravitational acceleration. ***F_u_****_,surf_* represents the surface tension force with the Marangoni effect, given by:

|  | $\vec{\boldsymbol{F}_{\boldsymbol{u,surf}}}=\sigma\kappa\delta\vec{n}+\left( \nabla\sigma-\left( \nabla\sigma\cdot\vec{\boldsymbol{n}} \right)\vec{\boldsymbol{n}} \right)\delta$ | Equation (S15) |
| --- | --- | --- |

where $\sigma$=$\sigma$(*T*) is the temperature-dependent surface tension coefficient, $\kappa$ is the local surface curvature and $\vec{\boldsymbol{n}}$ is the unit vector normal to the surface. $\delta$ is non-zero only on the surface to detect the surface. *Q_T_* represents heat transport, including heat conduction by Fourier’s law, viscous work, latent heat for phase change and radiation:

|  | $Q_{T}=\frac{1}{\rho c_{p}}\left[ \nabla\cdot\left( \lambda\nabla T \right)+\frac{\partial\tau_{ij}u_{i}}{\partial x_{j}}-\frac{D\rho\Delta h}{Dt} \right]+\varepsilon\sigma_{SB}(T^{4}-{T_{0}}^{4})\left\vert\nabla\varphi\right\vert$ | Equation (S16) |
| --- | --- | --- |

where Δ*h* is the latent heat. $\varphi$ ($0\leq\varphi\leq1$) is a function to identify the surface. Radiation was included on the heated metal surface identified by the gradient of $\left| \nabla\varphi\right|$, with the Stefan-Boltzmann constant, $\sigma$*_SB_*, and emissivity, $\varepsilon$. The laser power was defined as the Gaussian profile, and the laser ray was tracked by the ray tracing method. The physical properties such as viscosity and thermal conductivity were retrieved from Shinjo *et al*.^22^

Liquid/gas and solid/gas surface shapes were captured by the combined Level-Set/Volume-Of-Fluid (CLSVOF) method to ensure shape accuracy and volume conservation. The level-set function (signed distance function from the surface) was governed by:

|  | $\frac{\partial F}{\partial t}+\left( \vec{\boldsymbol{u}}\cdot\nabla\right)F=-\left\vert\nabla F \right\vert s$ | Equation (S17) |
| --- | --- | --- |

where $s$ is the surface regression speed due to evaporation. The colour function $\varphi$ was linked to *F* as a Heaviside function. The density at the melt pool surface, for example, was calculated as $\rho=\left( 1-\varphi\right)\rho_{G}+\varphi\rho_{L}$ where $\rho_{L}$ and $\rho_{G}$ are the liquid and gas density, respectively.

In **Supplementary Fig. 8,** we show a schematic of the Knudsen-vapour layer conditions used in the Ki *et al.*/Wang *et al.* simulations.^3,23^ For brevity, in the main text we only describe the subsonic case, whilst the full derivation is given here.

The jump conditions for the Ki *et al.*/Wang *et al.* model, without the subsonic assumption, can be described as: ^3^

|  | $\frac{T_{v}}{T_{s}}=\left( \sqrt{1+\pi\left( \frac{\gamma_{v}-1}{\gamma_{v}+1}\frac{\tilde{m}}{2} \right)^{2}}-\sqrt{\pi}\frac{\gamma_{v}-1}{\gamma_{v}+1}\frac{\tilde{m}}{2} \right)^{2}$ | Equation (S18) |
| --- | --- | --- |
|  | $\frac{\rho_{v}}{\rho_{s}}=\sqrt{\frac{T_{s}}{T_{v}}}\left[ \left( \tilde{m}^{2}+\frac{1}{2} \right)e^{\tilde{m}^{2}}\mathrm{erfc}\left( \tilde{m} \right)-\frac{\tilde{m}}{\sqrt{\pi}} \right]+\frac{1}{2}\frac{T_{s}}{T_{v}}\left[ 1-\sqrt{\pi}\tilde{m}e^{\tilde{m}^{2}}\mathrm{erfc}\left( \tilde{m} \right) \right]$ | Equation (S19) |
|  | $\frac{p_{v}}{p_{a}}=1+\gamma_{a}M_{v}\frac{a_{v}}{a_{a}}\left( \frac{\gamma_{a}+1}{4}M_{v}\frac{a_{v}}{a_{a}}+\left[ 1+\left( \frac{\gamma_{a}+1}{4}M_{v}\frac{a_{v}}{a_{a}} \right)^{2} \right]^{1/2} \right)$ | Equation (S20) |

The subscripts correspond to the circled letters in **Supplementary Fig. 8**. We can adapt to the nondimensional number as:

|  | $\tilde{m}=\frac{u_{v}}{\sqrt{2R_{v}T_{v}}}=\sqrt{\frac{\gamma_{v}}{2}}M_{v}$ | Equation (S21) |
| --- | --- | --- |

where $u_{v}$ is the velocity, $\gamma_{v}$ is the ratio of specific heats. $R_{v}$ is the gas constant (the universal gas constant divided by the molar mass ($R_{v}=\frac{\tilde{R}}{M}$), $M_{v}$ is the Mach number and $a$ is the speed of sound. If the Mach number is less than 1, the derivation continues as described in the **Methods**.

# Supplementary References

1. Flint, T. F., Scotti, L., Basoalto, H. C. & Smith, M. C. A thermal fluid dynamics framework applied to multi-component substrates experiencing fusion and vaporisation state transitions. *Commun Phys* **3**, 196 (2020).

2. Andersson, J. O., Helander, T., Höglund L, Shi P.F & Sundman B. Thermo-Calc and DICTRA, Thermo-Calc database TCNI12, Version 2025a. Preprint at https://thermocalc.com/support/documentation/ (2002).

3. Ki, H., Mohanty, P. S. & Mazumder, J. Modeling of laser keyhole welding: Part I. Mathematical modeling, numerical methodology, role of recoil pressure, multiple reflections, and free surface evolution. *Metall Mater Trans A Phys Metall Mater Sci* **33**, 1817–1830 (2002).

4. Wang, L., Zhang, Y. & Yan, W. Evaporation Model for Keyhole Dynamics during Additive Manufacturing of Metal. *Phys Rev Appl* **14**, 064039 (2020).

5. Bitharas, I. *et al.* The interplay between vapour, liquid, and solid phases in laser powder bed fusion. *Nat Commun* **13**, 2959 (2022).

6. Guo, D. *et al.* Correlative spatter and vapour depression dynamics during laser powder bed fusion of an Al-Fe-Zr alloy. *International Journal of Extreme Manufacturing* **6**, 055601 (2024).

7. Kramida A, Olsen, K. & Ralchenko, Y. NIST Atomic Spectra Database (ver. 5.9). *National Institute of Standards and Technology, Gaithersburg, MD* Preprint at https://physics.nist.gov/PhysRefData/ASD/LIBS/libs-form.html (2022).

8. Palleschi, V. Laser-induced breakdown spectroscopy: principles of the technique and future trends. *ChemTexts* **6**, 18 (2020).

9. Zhang, T., Tang, H. & Li, H. Chemometrics in laser-induced breakdown spectroscopy. *J Chemom* **32**, 2983 (2018).

10. Liu, K. *et al.* Interference correction for laser-induced breakdown spectroscopy using a deconvolution algorithm. *J Anal At Spectrom* **35**, 762–766 (2020).

11. Guezenoc, J. *et al.* Variable selection in laser-induced breakdown spectroscopy assisted by multivariate analysis: An alternative to multi-peak fitting. *Spectrochim Acta Part B At Spectrosc* **152**, 6–13 (2019).

12. Zhang, B., Yu, H., Sun, L., Xin, Y. & Conga, Z. A method for resolving overlapped peaks in laser-induced breakdown spectroscopy (LIBS). *Appl Spectrosc* **67**, 1087–1097 (2013).

13. Yang, W., Li, B., Zhou, J., Han, Y. & Wang, Q. Continuous-wavelet-transform-based automatic curve fitting method for laser-induced breakdown spectroscopy. *Appl Opt* **57**, 7526–7532 (2018).

14. Lu, S. *et al.* Analysis of salt mixture contamination on insulators via laser-induced breakdown spectroscopy. *Applied Sciences (Switzerland)* **10**, 2617 (2020).

15. Bennett, B. N., Martin, M. Z., Leonard, D. N. & Garlea, E. Calibration curves for commercial copper and aluminum alloys using handheld laser-induced breakdown spectroscopy. *Appl Phys B* **124**, 42 (2018).

16. Cavalcanti, G. H. *et al.* One-point calibration for calibration-free laser-induced breakdown spectroscopy quantitative analysis. *Spectrochim Acta Part B At Spectrosc* **87**, 51–56 (2013).

17. Hu, Z. *et al.* Self-absorption correction method for one-point calibration laser-induced breakdown spectroscopy. *Opt Lett* **48**, 1–4 (2023).

18. Liu, J. & Wen, P. Metal vaporization and its influence during laser powder bed fusion process. *Mater Des* **215**, 110505 (2022).

19. Bird, E. & Liang, Z. Transport phenomena in the Knudsen layer near an evaporating surface. *Phys Rev E* **100**, (2019).

20. Shinjo, J. *et al.* Physics-based thermal-chemical-fluid-microstructure modelling of in-situ alloying using additive manufacturing: Composition-microstructure control. *Addit Manuf* **64**, (2023).

21. Zhang, K. *et al.* Pore evolution mechanisms during directed energy deposition additive manufacturing. *Nat Commun* **15**, 1715 (2024).

22. Shinjo, J. & Panwisawas, C. Digital materials design by thermal-fluid science for multi-metal additive manufacturing. *Acta Mater* **210**, 116825 (2021).

23. Wang, J. *et al.* Numerical simulation of laser-induced plasma in background gas considering multiple interaction processes. *Plasma Science and Technology* **23**, (2021).
